# Spon1^+^ inflammatory monocytes promote collagen remodeling and lung cancer metastasis through lipoprotein receptor 8 signaling

**DOI:** 10.1172/jci.insight.168792

**Published:** 2024-05-08

**Authors:** Kristina M. Whately, Nisitha Sengottuvel, Lincy Edatt, Sonal Srivastava, Allison T. Woods, Yihsuan S. Tsai, Alessandro Porrello, Matthew P. Zimmerman, Aaron C. Chack, Stuart R. Jefferys, Gabriella Yacovone, Dae Joong Kim, Andrew C. Dudley, Antonio L. Amelio, Chad V. Pecot

**Affiliations:** 1 UNC Lineberger Comprehensive Cancer Center and; 2 Department of Genetics and Molecular Biology, University of North Carolina at Chapel Hill, Chapel Hill, North Carolina, USA.; 3 Department of Tumor Microenvironment and Metastasis, H. Lee Moffitt Cancer Center and Research Institute, Tampa, Florida, USA.; 4 Department of Cell Biology and Physiology and; 5 Department of Genetics, University of North Carolina at Chapel Hill, Chapel Hill, North Carolina, USA.; 6 Department of Microbiology, Immunology, and Cancer Biology and; 7 UVA Comprehensive Cancer Center, The University of Virginia, Charlottesville, Virginia, USA.; 8 Department of Head and Neck-Endocrine Oncology, H. Lee Moffitt Cancer Center and Research Institute, Tampa, Florida, USA.; 9 Division of Oncology and; 10 RNA Discovery Center, University of North Carolina at Chapel Hill, Chapel Hill, North Carolina, USA.

**Keywords:** Immunology, Oncology, Collagens, Lung cancer, Monocytes

## Abstract

Lung cancer is the leading cause of cancer-related deaths in the world, and non–small cell lung cancer (NSCLC) is the most common subset. We previously found that infiltration of tumor inflammatory monocytes (TIMs) into lung squamous carcinoma (LUSC) tumors is associated with increased metastases and poor survival. To further understand how TIMs promote metastases, we compared RNA-Seq profiles of TIMs from several LUSC metastatic models with inflammatory monocytes (IMs) of non–tumor-bearing controls. We identified *Spon1* as upregulated in TIMs and found that *Spon1* expression in LUSC tumors corresponded with poor survival and enrichment of collagen extracellular matrix signatures. We observed SPON1^+^ TIMs mediate their effects directly through LRP8 on NSCLC cells, which resulted in TGF-β1 activation and robust production of fibrillar collagens. Using several orthogonal approaches, we demonstrated that SPON1^+^ TIMs were sufficient to promote NSCLC metastases. Additionally, we found that *Spon1* loss in the host, or *Lrp8* loss in cancer cells, resulted in a significant decrease of both high-density collagen matrices and metastases. Finally, we confirmed the relevance of the SPON1/LRP8/TGF-β1 axis with collagen production and survival in patients with NSCLC. Taken together, our study describes how SPON1^+^ TIMs promote collagen remodeling and NSCLC metastases through an LRP8/TGF-β1 signaling axis.

## Abstract 2

Inflammatory monocyte SPON1 acts on LRP8-expressing cancer cells to activate TGF&beta;, which in-turn triggers cancer cell mediated collagen remodeling to promote NSCLC metastases.

## Graphical abstract

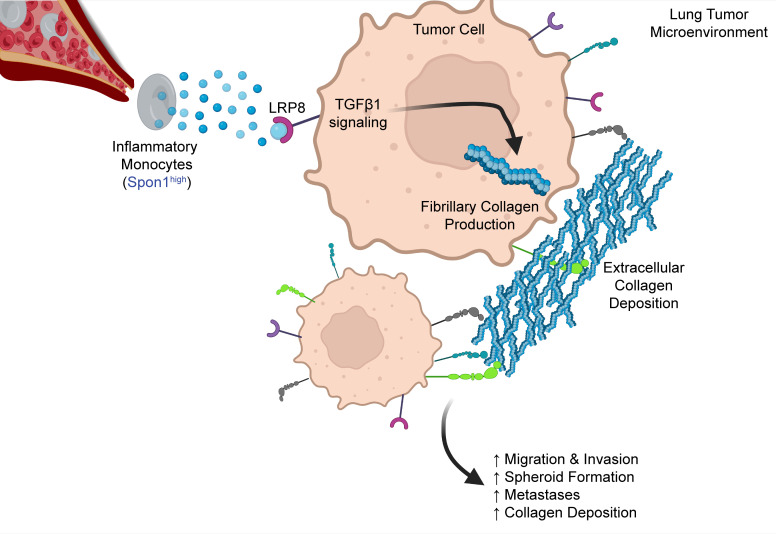

## Introduction

Lung cancer causes the most deaths in both men and women with cancer, taking more lives than colon, breast, and liver cancers combined (1). The probability of developing lung cancer for any individual is 1 in 16 for men and 1 in 17 for women (1). While these odds include the general population, the risk increases drastically for individuals with a smoking history (2). The 2 major subtypes of lung cancer are small cell lung cancer and non–small cell lung cancer (NSCLC). NSCLC is further divided into lung adenocarcinoma (LUAD) and lung squamous cell carcinoma (LUSC). For patients with NSCLC, the 5-year relative survival rate was 22% across all stages between 2011 and 2017 (1). Although immune checkpoint inhibitors (ICI) have significantly improved both progression-free and overall survival, revolutionizing the treatment of NSCLC (3, 4), only about 20% of unselected patients with LUSC respond to these therapies (5–7). Various combination therapies such as chemotherapy and ICI therapy (e.g., anti-CTLA4 and anti–PD-1) can increase the percentage of patients who can benefit from these inhibitors, but durable responses to these combinations are limited (8). Therefore, further evaluation of NSCLC progression is needed to establish novel therapeutics for patients.

In addition to what is known about the unfavorable conditions produced by a PD-1 ligand (PD-L1) negative status for anti–PD-1 therapies, profiling the immune cell populations of nonresponders to anti–PD-1 inhibition has suggested that the infiltration of immune suppressive cell populations like Tregs and myeloid cells may be responsible for resistance (9). Myeloid cells constitute a high proportion of the infiltrating cells in tumors, and preclinical models suggest that these cells have an important role in promoting metastases and poor patient survival across several cancer types (10). For example, bulk RNA-Seq of sorted populations of myeloid cells in immunocompetent genetically engineered mouse models (GEMM) of LUAD showed that tumor-associated neutrophils (TANs) cause T cell suppression by arginine depletion (11). Arginase inhibition was able to successfully prevent tumor growth and promoted an antitumor immune microenvironment in an immunocompetent LUAD mouse model. Another group studying transdifferentiation of LUAD into LUSC found Sox2-driven recruitment of TANs to the periphery of tumors, regardless of their subtype (12). A different study found that dual depletion of Lkb1 and Pten resulted in a LUSC histology and recruitment of TANs to the tumor, while Kras mutations produced LUAD histologies and were more characterized by macrophage signatures (13). A key myeloid driver of several protumorigenic phenotypes is tumor-associated macrophages (TAMs). TAMs are made up of a polarized M2 phenotype when they infiltrate tumor tissues, and they promote tumor formation and metastases through suppressing lymphoid cells and alter the TME by production of growth factors like FGF, TGF-β, and VEGF to promote angiogenesis and connective tissue degradation (14, 15). TAMs have also been known to modify the ECM by creating a profibrotic microenvironment and degrading collagen in the TME (16, 17). Thus, TAMs have been shown to promote tumorigenesis by leading to senescence and to promote increased invasion and metastasis (18–20).

Previously, tumor inflammatory monocytes (TIMs) were often regarded as inactive precursors of TAMs (21). However, high monocyte counts in the peripheral blood correlated inversely with patient survival in patients with pancreatic cancer with resected tumors (22). TIMs are usually identified with the same markers as monocytic myeloid–derived suppressor cells (MDSCs: CD11b^+^Ly6C^hi^Ly6G^–^) (23). TIMs preferentially migrate to metastatic lesions over primary tumors (24), and several studies have started to explore their roles in the TME beyond T cell suppression, such as extracellular matrix (ECM) remodeling via fibrin cross-linking, wound-healing, and promotion of tumor angiogenesis (24–27). Recent evidence has shown unique roles specific to TIMs in NSCLC. LUAD tumor cells have been shown to recruit CD14^+^ monocytes, and patients expressing high levels of CD14^+^ cells in their tumors were found to have worse prognosis when compared with their CD14^mid^ and CD14^lo^ counterparts (28). Immunogenomic analysis of The Cancer Genome Atlas (TCGA) has revealed that approximately half of LUSC tumors are characterized by dense infiltration of TIMs and poor survival and has shown that CCL2-mediated recruitment of TIMs is necessary and sufficient to promote lung cancer metastases (25). Using a comprehensive proteogenomic approach of resected LUSC tumors, another group found that “inflamed” subtypes of LUSC had higher recruitment of monocytes associated with significantly higher expression of the ICI marker PD-1 (29). While the importance of TIMs in NSCLC is beginning to be appreciated, the mechanisms by which TIMs promote lung cancer progression remain poorly understood.

Here, by integrating bioinformatic approaches with in vitro and in vivo modeling of the TME, we demonstrate that F-Spondin–expressing (SPON1-expressing) TIMs promote fibrillar collagen production and metastases through a LRP8/TGF-β1 signaling axis in cancer cells. Taken together, these findings demonstrate a previously unappreciated role of how TIMs co-opt cancer cells to remodel collagen and reveal a potentially new therapeutic vulnerability in a difficult-to-treat disease.

## Results

### SPON1 is highly expressed in tumor-infiltrating inflammatory monocytes.

**Figure 1:**
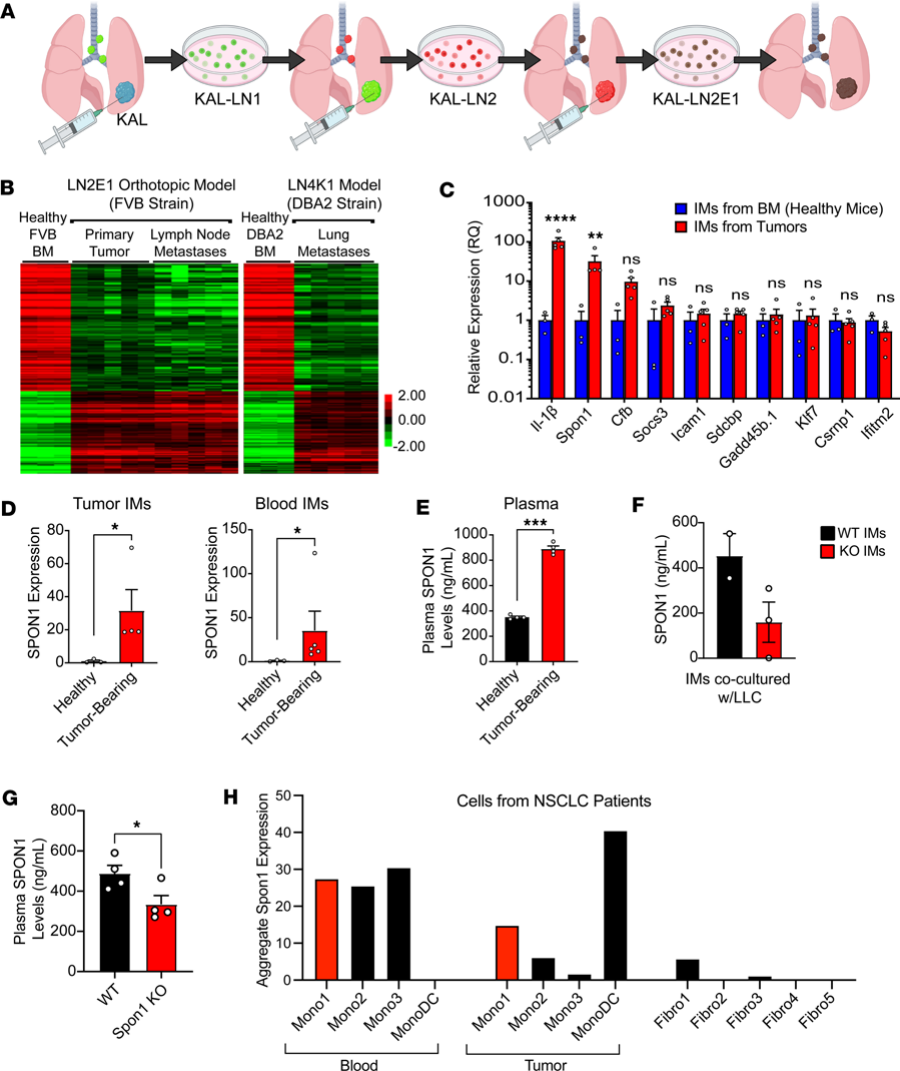
Identifying Spon1 as an important mediator of LUSC disease progression. (**A**) Model showing the development of the KAL-LN2E1 metastatic LUSC cell line by in vivo passaging. (**B**) RNA-Seq data of genes that are differentially expressed in TIMs from both the LN2E1 and LN4K1 tumor models (across the 3 experimental conditions) when compared with their respective host strain BM-derived IMs (used as baselines). (**C**) Relative expression of top 10 genes with highest hazard ratios found to be overexpressed in TIMs by qPCR. (**D**) *Spon1* expression in healthy versus tumor-bearing IMs from tumors and blood. (**E**) Plasma SPON1 concentrations (ng/mL) in healthy versus tumor bearing mice. (**F**) measure of SPON1 released from WT versus *Spon1^–/–^* IMs. (**G**) SPON1 levels as seen in plasma taken from WT versus *Spon1^–/–^* mice with orthotopic LLC tumors at day 19. (**H**) Total *SPON1* expression for each cell type from human NSCLC single cell samples. ****P < 0.001, ***P < 0.001, **P < 0.01, *P < 0.05. Data are shown as the mean ± SEM incorporating biological and technical replicate samples. Two-tailed Student’s *t* test for 2-group comparisons.

To evaluate how TIMs (CD45^+^SiglecF^–^CD11b^+^Ly6G^–^Ly6C^hi^) may promote lung cancer metastasis, we utilized 2 models generated in our lab to study gene expression profiles of TIMs. We used primary tumors and lymph node metastases from the highly metastatic KAL-LN2E1 (LN2E1) model, which was derived from the KAL LUSC parental cell line that grew in FVB mice (25). We also used an experimental metastases model with our KLN-LN4K1 (LN4K1) model, which was derived from the parental cell line KLN-205 (25). Using inflammatory monocytes (IMs) sorted from LN2E1 primary tumors and lymph node metastases (Figure 1A), and using LN4K1 experimental metastases, we compared RNA-Seq profiles of TIMs with IMs harvested from the BM (BM IMs) of non–tumor-bearing mice. Using stringent thresholds, we found 283 differentially expressed genes (DEGs) in TIMs that were consistently overexpressed in primary tumors, lymph node metastases, and distant lung metastases when compared with IMs from healthy BM in the same strain of mice (Figure 1B). From the list created, we selected the top 10 upregulated genes most strongly associated with poor survival in the LUSC TCGA data set for further validation (Figure 1C and Supplemental Table 1; supplemental material available online with this article; https://doi.org/10.1172/jci.insight.168792DS1). In a separate validation experiment, we confirmed that *Il-1β*, *Spon1*, and *Cfb* showed significantly increased expression in TIMs in the LN2E1 model when compared with IMs from the BM of non–tumor-bearing mice. *IL-1β* is known to be expressed in TIMs and has been studied in NSCLC (30); however, recent clinical trials using an IL-1β targeting antibody (Canakinumab) failed to show improved NSCLC outcomes (31–35). Because SPON1-expressing TIMs have never been studied, to our knowledge, we sought to understand how SPON1 in the TME may support NSCLC progression and poor survival. Compared with non–tumor-bearing mice, we observed that IMs isolated from tumors and circulating IMs in tumor-bearing mice showed overexpression of *Spon1* (Figure 1D). We also evaluated SPON1 levels in the serum and observed a significant increase in tumor-bearing mice (Figure 1E). Consistent with SPON1 promoting lung cancer progression, LUSC tumors expressing high levels of *SPON1* had decreased overall survival (Supplemental Figure 1A). Additionally, in earlier stages of LUSC, elevated *SPON1* expression levels were associated with decreased overall survival (Supplemental Figure 1, B and C).

To determine if SPON1 can be secreted from IMs, we cocultured BM derived IMs from WT or *Spon1*^–/–^ mice with Lewis Lung Carcinoma (LLC) cells. An ELISA of the conditioned media 5 days later showed increased SPON1 secretion from WT IMs (Figure 1F). We confirmed that the lung cancer cells (LN2E1, LN4K1, and LLC) had low expression of *Spon1* compared with IMs isolated from BM or TIMs isolated from LLC tumors (Supplemental Figure 1, D and E). When compared with WT mice, *Spon1*^–/–^ mice orthotopically injected with LLC cells showed reduced serum levels of SPON1 (Figure 1G). Using a publicly available single-cell RNA-Seq data set from patients with NSCLC (36), we evaluated myeloid subsets for *SPON1* expression. Interestingly, we found that multiple Mono cell subsets express *SPON1*; this subset includes the inflammatory monocyte population, Mono1 (Figure 1H). Consistent with our mouse model, the Mono1 population in blood and tumor samples of patients with NSCLC showed elevated *SPON1* expression. Taken together, these results suggest that TIMs are a source of SPON1 within the TME and that SPON1^+^ TIMs may promote NSCLC progression.

### SPON1^hi^ patients show high levels of EMT processes.

**Figure 2:**
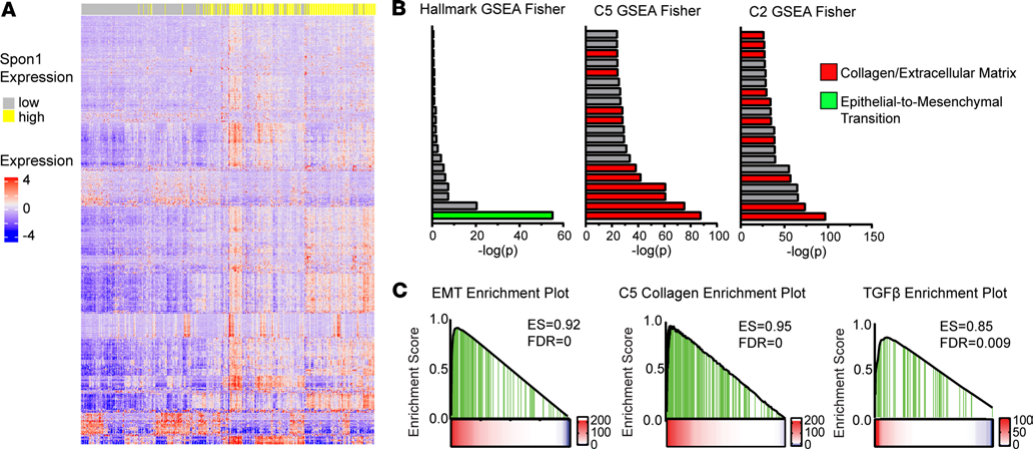
*SPON1*^hi^ patients show enrichment of EMT and collagen remodeling gene sets. (**A**) Differentially expressed genes between *SPON1*^hi^ and *SPON1*^lo^ patients. GSEA following Fisher’s Exact Test. (**B**) Top 20 most highly significant gene sets enriched in the Hallmark, C5, and C2 data sets. (**C**) Specific enrichment plots for EMT (Hallmark), Collagen (C5), and TGF-β (Hallmark) pathways.

To better understand the mechanism underlying the poor prognosis we observed in *SPON1*^hi^ patients, we used TCGA data to examine DEGs between patients below (*SPON1*^lo^) and above (*SPON1*^hi^) the median expression threshold (Figure 2A). Using Gene set enrichment analysis (GSEA), we found that *SPON1*^hi^ patients showed highly significant enrichment in epithelial-to-mesenchymal transition (EMT) and collagen/ECM-related gene sets (Figure 2B and Supplemental Table 2). In the Hallmarks gene sets curated, EMT was the most significantly enriched gene set (FDR *q* = 0, *P* = 4.12 × 10^–56^) (37). Additionally, many gene sets were enriched for collagen and ECM pathways. In the C5 collection of gene sets, “collagen containing ECM” (FDR *q* = 0.012, *P* = 1.81 × 10^–76^) followed the ECM in most significantly enriched gene sets in *SPON1*^hi^ patients. Among the C2 gene sets, the NABA and NABA Core Matrisomes were the 2 most highly enriched gene sets, which represents a collection of ECM and associated proteins that notably include 36 collagen genes (38). Consistent with these patterns, we saw that enrichment plots for the EMT (Hallmark), collagen (C5) and TGF-β (Hallmark) pathways were all highly enriched (Figure 2C and Supplemental Table 3). To begin understanding how SPON1 may be related to these findings, we compiled a list of 31 genes that overlapped between the most highly enriched gene sets (Supplemental Figure 2). To evaluate how SPON1 may have a role in promoting cancer progression, we further selected the top 11 genes from this initial list with the highest hazard ratios, with a score above 1 indicating worse survival in patients with LUSC (Supplemental Table 4). Notably, almost half of these genes were fibrillar collagens, including *COL1A1*, *COL1A2*, *COL3A1*, *COL5A1*, and *COL11A1*, which are known to promote tumorigenesis by forming tightly aligned fibrils that increase the ECM stiffness, allowing for increased cell invasion (39–41). Other collagens and matrix remodeling proteins in this list included *COL12A1*, *COMP*, *GREM1*, *MMP2*, *POSTN*, and *THBS2*. These data suggest that SPON1^+^ TIMs may support lung cancer progression through EMT and collagen remodeling.

### SPON1^+^ inflammatory monocytes promote spheroid formation and collagen production.

**Figure 3:**
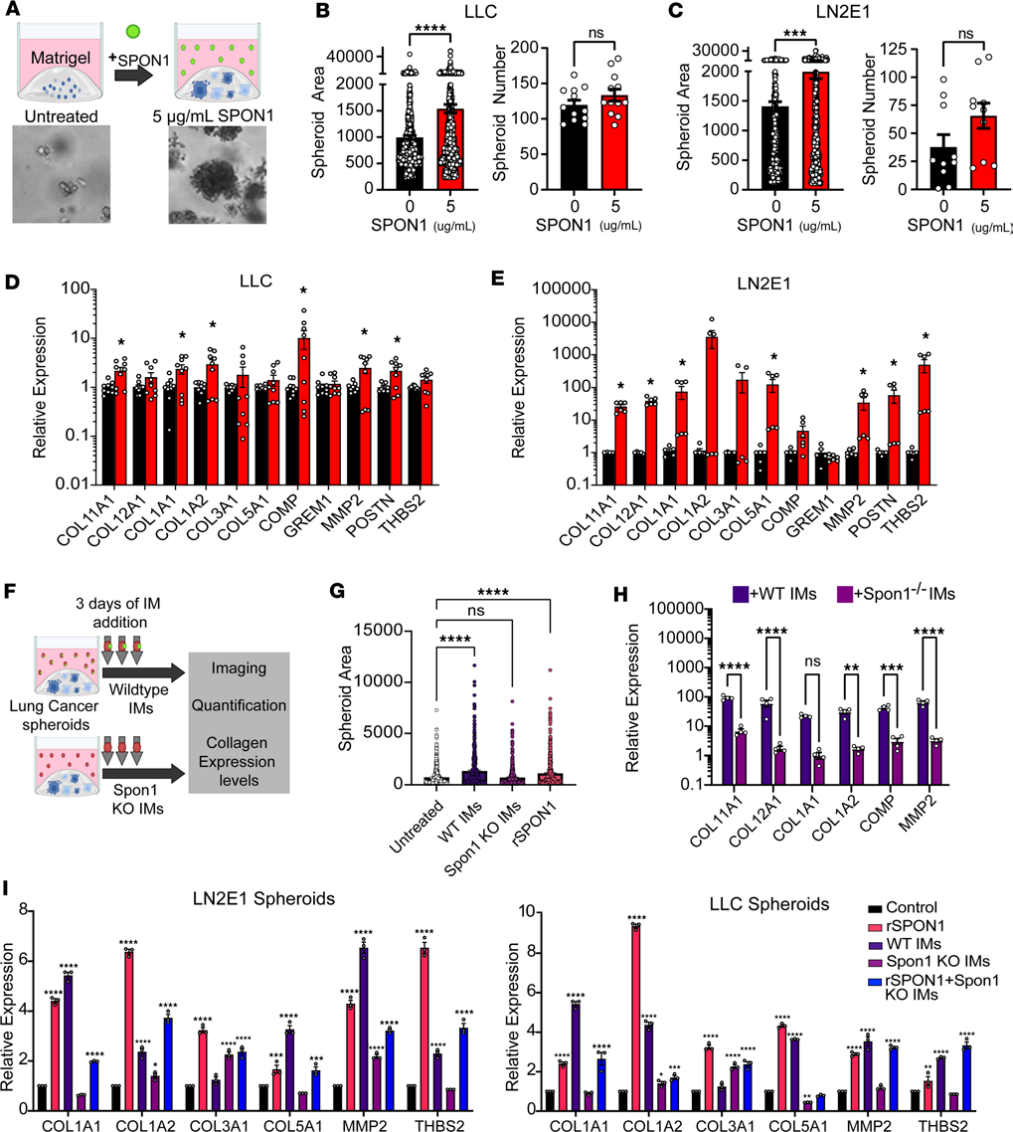
Spon1 increases fibrillar collagen and collagen remodeling gene expression in cancer cells. (**A**) Schematic and representative LN2E1 spheroid formation assay in Matrigel with recombinant SPON1 treatment. (**B** and **C**) Spheroid area and numbers for LLC and spheroid area and numbers for LN2E1 with and without recombinant murine SPON1 treatment (5 μg/mL). (**D** and **E**) Collagen gene expression with SPON1 treatment in LLC spheroids and in LN2E1 spheroids. (**F**) Schematic of WT or *Spon1^–/–^* IM coculture with LN2E1 or LLC spheroids. (**G**) Spheroid area and collagen gene expression with WT or *Spon1^–/–^* IM coculture with LN2E1 spheroids. (**H**) Collagen gene expression with WT or *Spon1^–/–^* IM coculture with LLC spheroids. (**I**) Collagen gene expression of LN2E1 and LLC spheroids with recombinant SPON1, WT IMs, *Spon1^–/–^* IMs, or recombinant SPON1 plus *Spon1^–/–^* IMs. ****P < 0.001, ***P < 0.001, **P < 0.01, *P < 0.05. Data are shown as the mean ± SEM incorporating biological and technical replicate samples. Two-tailed Student’s *t* test for 2-group comparisons; 1-way ANOVA test for multiple comparisons.

To test the direct effects of SPON1 on lung cancer promotion and collagen production, we treated several NSCLC lines with recombinant SPON1 in vitro. Notably, SPON1 treatment resulted in no differences in cell proliferation rates (Supplemental Figure 3). Since EMT pathways were enriched in *SPON1*^hi^ patients, we evaluated rates of migration and invasion in the same cell lines. The LN4K1 model showed significantly increased migration and invasion with SPON1 treatment, while LN2E1 only showed modest increases (Supplemental Figure 4). These findings are consistent with another study showing treatment of osteosarcoma cell lines with SPON1 increased cell migration and invasion, whereas *Spon1* knockdown reduced these phenotypes (42). Next, we evaluated NSCLC cells grown in spheroids in the presence or absence of recombinant SPON1 and evaluated for spheroid area and numbers (Figure 3A). We saw a significant increase in spheroid area and a modest increase in spheroid numbers for both LUAD (LLC) (Figure 3B) and LUSC (LN2E1) (Figure 3C) cell lines with SPON1 treatment. Furthermore, although fibrillar collagens are typically thought to arise from cancer-associated fibroblasts (CAFs) (43), we observed substantial intrinsic upregulation of many collagen genes in cancer cells in response to recombinant SPON1 treatment (Figure 3, D and E, and Supplemental Figure 5). To evaluate whether SPON1 secreted from IMs could have a similar effect as recombinant SPON1, we isolated BM IMs from either WT C57BL/6 or *Spon1*^–/–^ mice and cocultured them with cancer cell spheroids for 3 consecutive days (Figure 3F). We observed significant increases in spheroid formation with WT IM addition (*P* < 0.0001), which phenocopied recombinant SPON1. The addition of *Spon1*^–/–^ IMs, however, did not lead to any increase in spheroid area (Figure 3G). Additionally, compared with spheroids cocultured with WT IMs, spheroids cocultured with *Spon1*^–/–^ IMs possessed significantly reduced expression of many collagen genes (Figure 3H). Furthermore, recombinant SPON1 can restore the induced expression of collagen genes when cocultured with *Spon1*^–/–^ IMs (Figure 3I). Together, our findings demonstrate that IMs produce and secrete sufficient SPON1 to promote spheroid formation, and unexpectedly, SPON1 can directly induce large amounts of collagen production in NSCLC spheroids.

### SPON1 loss abrogates NSCLC progression and collagen production.

**Figure 4:**
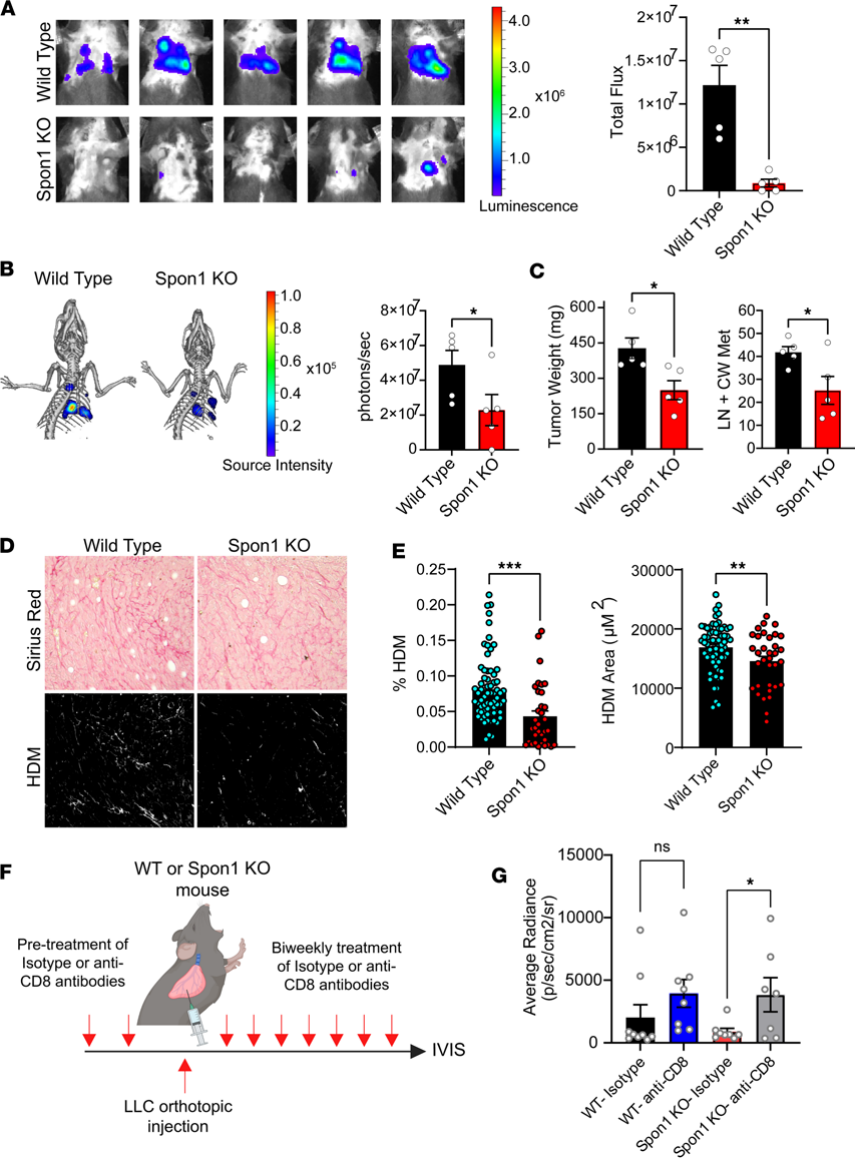
Reduced tumor formation and collagen production in the absence of Spon1. (**A**) IVIS imaging at 15 days from injection of LLC tumors into either WT or *Spon1^–/–^* mice. (**B**) Three-dimensional optical plus CT imaging at day 15 of LLC tumors in WT and *Spon1^–/–^* mice with higher resolution quantification. (**C**) Weight and counts of tumor burden from LLC tumors in WT and *Spon1^–/–^* mice. (**D**) Picrosirius red staining of FFPE tumors from WT and *Spon1^–/–^* mice. (**E**) Quantification of collagen read outs for high-density matrix (HDM). (**F**) Schematic of CD8 depletion experiment. (**G**) IVIS results at day 13 of LLC tumors in WT or *Spon1^–/–^* mice with Isotype or anti-CD8 antibody treatment. ***P < 0.001, **P < 0.01, *P < 0.05. Data are shown as the mean ± SEM incorporating biological and technical replicate samples. Two-tailed Student’s *t* test for 2-group comparisons. Total original magnification, ×20.

To evaluate the ability of SPON1 to modulate spontaneous metastasis, we orthotopically injected luciferase-labeled LLC (LLC-Luc) cells into the lungs of either WT or *Spon1*^–/–^ mice and monitored mice until either group reached a moribund endpoint. Using 2D optical IVIS and 3D computed tomography with IVIS Spectrum bioluminescent imaging (BLI), we observed that *Spon1*^–/–^ mice had significantly reduced disease burden (Figure 4, A and B). Consistent with our BLI findings, a cross-sectional necropsy showed a significant decrease in both aggregate weight and number of metastatic lesions in the *Spon1*^–/–^ mice compared with WT mice (Figure 4C). However, s.c. tumor growth of LLC tumors grew significantly faster in *Spon1*^–/–^ mice compared with WT (Supplemental Figure 4G). These findings demonstrate that loss of *Spon1* results in significantly reduced NSCLC metastatic progression, which is unrelated to its effects on tumor growth. Tumor sections from the necropsy samples were stained with Picrosirius red to assess relative collagen content. Using an automated image analysis tool to study tumor collagen matrices (44), we observed significantly higher levels of collagen content in the WT mice based on high-density matrix (HDM) percentage and area (Figure 4, D and E). To test the importance of the adaptive immune response in relation to SPON1, we depleted T cells using anti-CD8 antibodies and orthotopically injected LLC-Luc into WT and *Spon1*^–/–^ mice (Figure 4F). CD8 T cell depletion resulted in increased metastatic disease in both WT and *Spon1*^–/–^ mice (Figure 4G). This abrogation of the protective effect of *Spon1*^–/–^ suggests that T cells may play an important role in SPON1-mediated disease progression. These findings demonstrate that SPON1 within the TME supports metastases progression and collagen remodeling.

### SPON1^+^ TIMs are sufficient to promote lung cancer metastases.

**Figure 5:**
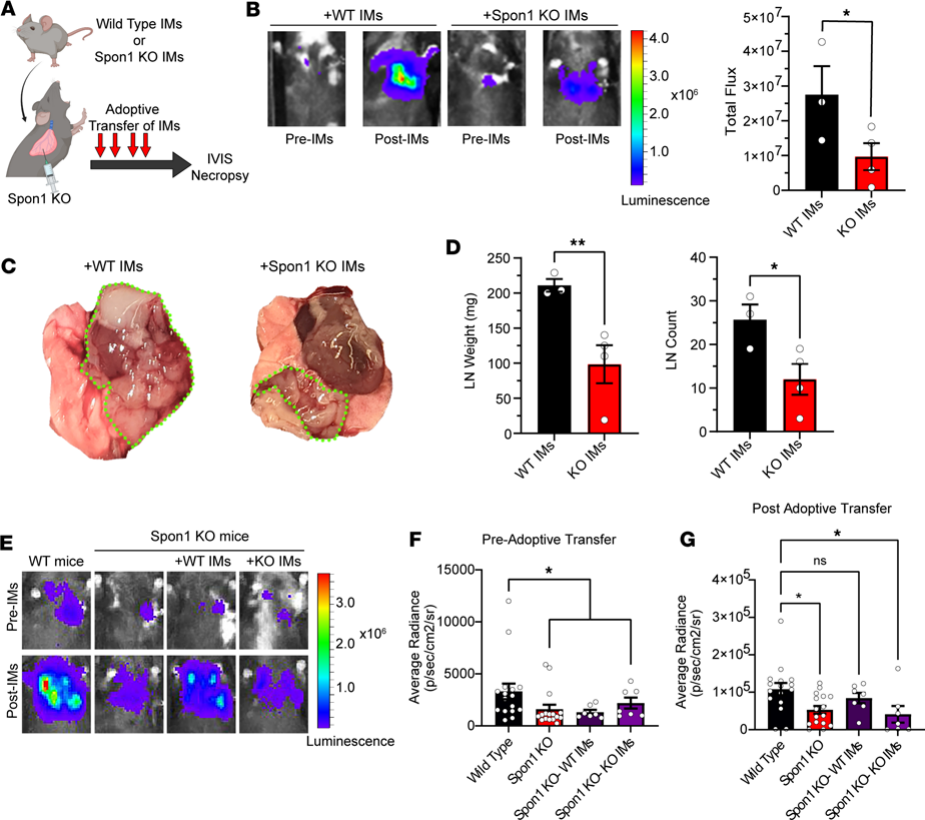
Phenotypic rescue of disease burden with restoration of Spon1^+^ TIMs. (**A**) Schematic of adoptive transfer experiment with either WT or *Spon1^–/–^* IMs infused into *Spon1^–/–^* mice orthotopically injected with LLC cells. Infusions took place on days 6, 8, 11, and 12 after injections. (**B**) IVIS results on day 14 after injection. *n* = 3. (**C**) Gross histology visualization of disease burden. (**D**) Lymph node tumor weight and counts on day 14 after injection. *n* = 3. (**E**) Representative IVIS images of WT and *Spon1^–/–^* mice with or without adoptive transfer of WT or KO IMs. (**F**) IVIS results on day 5 after injection. *n* = 7 each group. (**G**) IVIS results on day 13 after injection. *n* = 7 each group. **P < 0.01, *P < 0.05. Data are shown as the mean ± SEM incorporating biological and technical replicate samples. Two-tailed Student’s *t* test for 2-group comparisons; 1-way ANOVA test for multiple comparisons.

Although we observed significantly reduced metastases in the *Spon1*^–/–^ mice, it is possible that TIMs are not a sufficient source of SPON1 to promote metastases. To determine whether SPON1^+^ TIMs can promote NSCLC metastases, we orthotopically injected LLC-Luc into *Spon1*^–/–^ mice. Using BM IMs of either WT or *Spon1*^–/–^ mice, we adoptively transferred IMs on days 6, 8, 11, and 12 following LLC-Luc injection (Figure 5A). IVIS imaging on day 14 revealed that mice infused with WT IMs had significantly increased metastatic progression compared with the mice infused with IMs from donor *Spon1*^–/–^ mice (Figure 5B). These findings were corroborated based on necropsies showing significantly more metastases in the mice infused with WT IMs (Figure 5, C and D). To evaluate whether Spon1^+^ TIMs can fully rescue the loss of function seen in *Spon1*^–/–^ mice (Figure 4, A–C), we directly compared the effects of the adoptive transfers with WT and *Spon1*^–/–^ mice. Using IVIS imaging, before adoptive transfer, we observed significantly decreased tumor burden in all *Spon1*^–/–^ mice groups compared with WT mice (Figure 5, E and F). After adoptive transfer of WT and *Spon1*^–/–^ IMs in *Spon1*^–/–^ mice, we only observed rescue of disease burden with WT IMs in *Spon1*^–/–^ mice (Figure 5, E and G). These data demonstrate that SPON1^+^ TIMs are sufficient to promote NSCLC disease progression.

### SPON1 promotes NSCLC progression and collagen production through an LRP8/TGF-β1 signaling axis.

**Figure 6:**
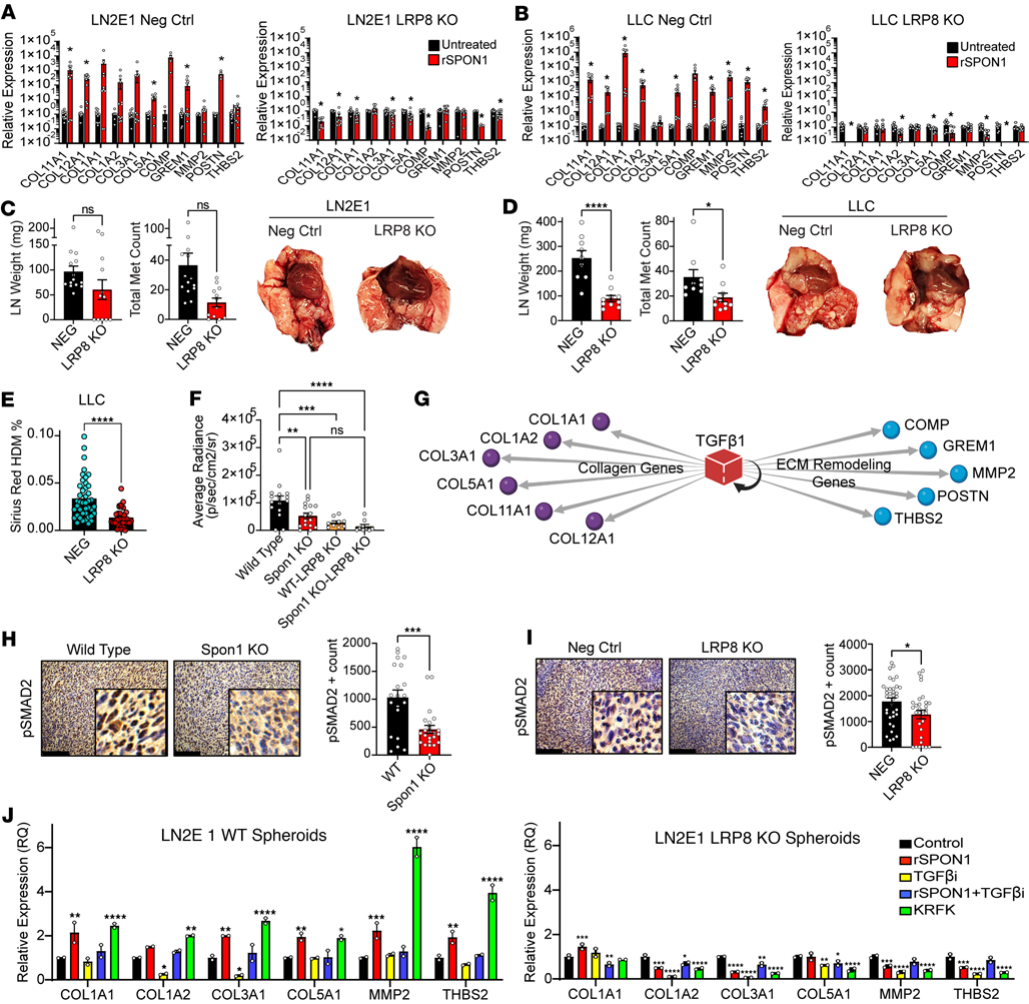
Spon1 mediates its effects on disease progression and collagen content via LRP8. (**A** and **B**) Collagen gene expression in both LN2E1 (**A**) and LLC (**B**) spheroids for negative control and LRP8 KO. (**C** and **D**) In vivo disease burden by tumor weight and counts in LN2E1 and LLC. (**E**) Collagen content in LLC shown by Sirius red high-density matrix levels. (**F**) In vivo IVIS results of LLC WT or LRP8-KO tumors in either WT mice or Spon1^–/–^ mice. *n* = 9 each group. (**G**) TGF-β1 as a top upstream regulator of the genes composing our Collagen Gene Signature. (**H** and **I**) Phospho-SMAD2 scoring of WT and *Spon1*^–/–^ LLC tumors and of negative control and LRP8-KO LLC tumors. Scale bars: 125 μM. (**J**) qPCR of Collagen and EMT genes of LN2E1 WT or LRP8-KO spheroids under treatment conditions of untreated, recombinant SPON1, TGF-βi (SB431542), recombinant SPON11^+^TGF-βi, and KRFK peptide. ****P < 0.001, ***P < 0.001, **P < 0.01, *P < 0.05. Data are shown as the mean ± SEM incorporating biological and technical replicate samples. Two-tailed Student’s *t* test for 2-group comparisons; 1-way ANOVA test for multiple comparisons.

SPON1 has previously been shown to bind to LRP8/ApoER2 and inhibit osteoclast differentiation and amyloid precursor protein processing (45, 46). To test whether the effects of SPON1 are dependent on LRP8 binding, we created *Lrp8* KOs in the LN2E1 and LLC cell lines (Supplemental Figure 6A). SPON1 treatment increased spheroid numbers in the LN2E1 model but not in the LLC models, and this effect was abrogated by *Lrp8* deletion (Supplemental Figure 6, B and C). Importantly, compared with control sgRNA lines, LN2E1 and LLC *Lrp8*-KO cells lost their ability to upregulate the collagen and matrix remodeling genes when treated with recombinant SPON1 treatment (Figure 6, A and B). Compared with controls, we also observed significantly reduced spontaneous metastases in both LN2E1 and LLC *Lrp8-*KO models in vivo (Figure 6, C and D) as well as a significant reduction in collagen HDMs (Figure 6E). Using the WT or *Lrp8-*KO LLC model in WT and *Spon1*^–/–^ mice, we found significantly decreased tumor burden by IVIS in WT mice with *Lrp8-*KO tumors, and a significantly diseased metastatic burden in the when both SPON1 and LRP8 are knocked out (Figure 6F). Together, these findings support that SPON1 mediates its effects on cancer metastasis and collagen production through its cognate receptor, LRP8.

Although our findings support that SPON1^+^ TIMs can upregulate diverse species of collagen production through tumor cell LRP8 expression, we then determined whether CAFs can also produce collagen via a SPON1/LRP8 axis. Using CAFs isolated from lung tumors of LSL-KRAS^G12D/+^ p53^fl/fl^ Lkb1^fl/fl^ mice, we observed similar LRP8 expression levels on CAFs compared with LLC cells (Supplemental Figure 6, D and E). Using publicly available single-cell RNA-Seq data from patients with NSCLC (36), we evaluated monocyte and fibroblast subsets for *SPON1* expression and found that fibroblast subsets generally have variable, and generally lower, levels of *SPON1* expression than TIMs (Mono1) (Figure 1H). Our findings for CAFs suggest low expression of SPON1 and the presence of LRP8 expression. To determine if the SPON1/LRP8 axis is similar in CAFs, we evaluated the collagen genes in CAFs treated with SPON1. Interestingly, we found a similar increase in collagen genes with SPON1 compared with the LLC spheroid model with SPON1 (Supplemental Figure 6F). This suggests that lung cancer CAFs also have a collagen gene response through SPON1/LRP8 signaling, but cancer cells are also capable of “mimicking” CAFs by using the SPON1/LRP8 axis to produce collagen.

To determine how SPON1/LRP8 signaling promotes collagen production in NSCLC cells, we analyzed the expression levels of the 11 genes in our collagen gene set after recombinant SPON1 treatment of spheroids. An upstream regulator analysis using Ingenuity Pathway Analysis showed TGF-β1 to be the most significant regulator for the 11 collagen and EMT genes upregulated in response to SPON1, classifying our collagen gene signature (Figure 6G and Supplemental Figure 7A). TGF-β1 has an established role in the promotion of matrix remodeling in the TME (47–49). Also, previous work has also shown that SPON1 can activate TGF-β1 in the context of cartilage metabolism (18). To evaluate whether SPON1 signaling through LRP8 can induce TGF-β1 activation, we evaluated several of our tumor models and spheroids for SMAD2 phosphorylation. Using our LLC model, compared with respective controls, we found significantly decreased levels of phospho-SMAD2 in tumors grown in *Spon1*^–/–^ mice (Figure 6H) or from *Lrp8*-KO lines (Figure 6I). Using LN2E1 spheroids with recombinant SPON1, we found an increase in phospho-SMAD2, which was abrogated when combined with a TGF-β inhibitor, SB431542 (Supplemental Figure 7, B and C). Similarly, we found decreased protein expression of phospho-SMAD2 in LLC spheroids cocultured with *Spon1*^–/–^ IMs (Supplemental Figure 7, D and E). To further confirm the SPON1-mediated activation of collagen and EMT genes through TGF-β activation, we assessed LN2E1 WT or *Lrp8*-KO spheroids with treatment of SPON1 or TGF-β inhibitor or activator (Figure 6J). In WT spheroids, we found these genes to be significantly increased with SPON1 treatment and with TGF-β activator, KRFK. Combined treatment of SPON1 and TGF-βi did not modulate gene expression any differently from the untreated spheroids, validating that SPON1-mediated effects are abrogated with TGF-β inhibition. In the *Lrp8*-KO spheroids, gene expression of certain genes is decreased in all treatment groups (Figure 6J). These results verify that SPON1 binding to LRP8 signals through TGF-β signaling to activate collagen and EMT genes.

**Figure 7:**
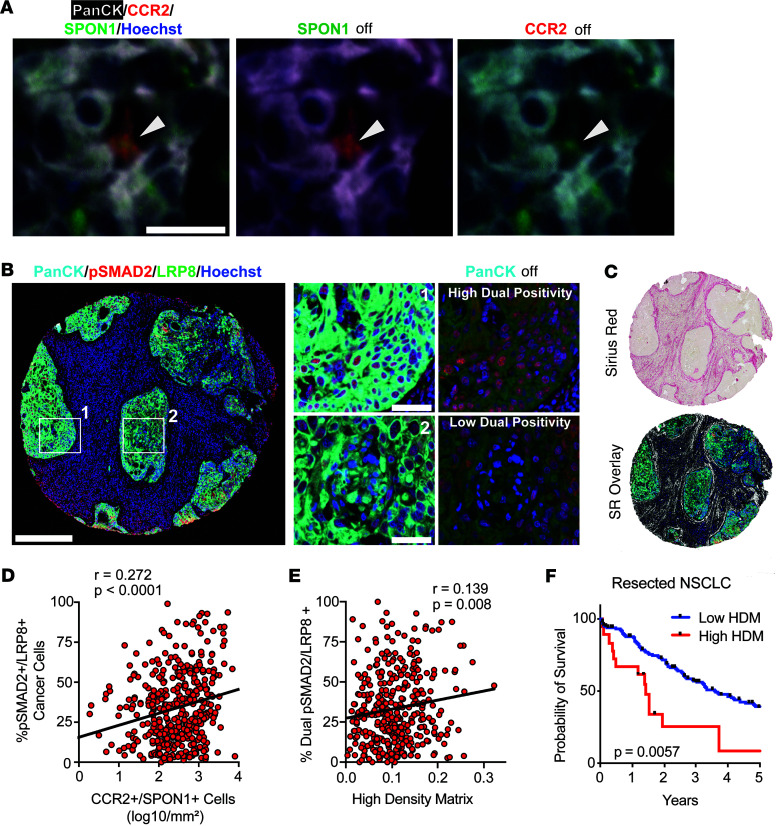
Patient NSCLC tumors show Spon1^+^ TIMs, and LRP8 expression on cancer cells and TGF-β1 signaling positively correlate with collagen expression, which leads to poor survival. (**A**) CCR2^+^SPON1^+^ staining identifies SPON1^+^ TIMs in tumor cell islets (PanCK^+^) in an LUAD tumor. PanCK, white; CCR2, red; SPON1, green; Hoechst (nuclear stain), blue. Scale bar: 25 μM. Arrowheads are pointing to a CCR2^+^SPON1^+^ TIM. (**B**) Immunofluorescence staining of LUSC tumors showing cancer cells (aqua) expressing LRP8 (green) with TGF-β1 activation (+pSMAD2/red). Scale bars: 300 μM (full core), 50 μM (insets). (**C**) Sirius red staining of the same core used in **B** and an overlay with positive sirius red staining shown in white. (**D** and **E**) Two-sided Pearson correlations of percentage of pSMAD2^+^LRP8^+^panCK^+^ cells with CCR2^+^SPON1^+^ cell density (*P* < 0.0001, *r* = 0.272) (**D**) and between pSMAD2^+^LRP8^+^panCK^+^ cells and high-density matrix indices based on Sirius red staining (**E**) (*P* = 0.008, *r* = 0.139). (**F**) Survival differences between patients expressing high and low HDM (*n* = 164 patients, *P* = 0.0057).

**Figure 8:**
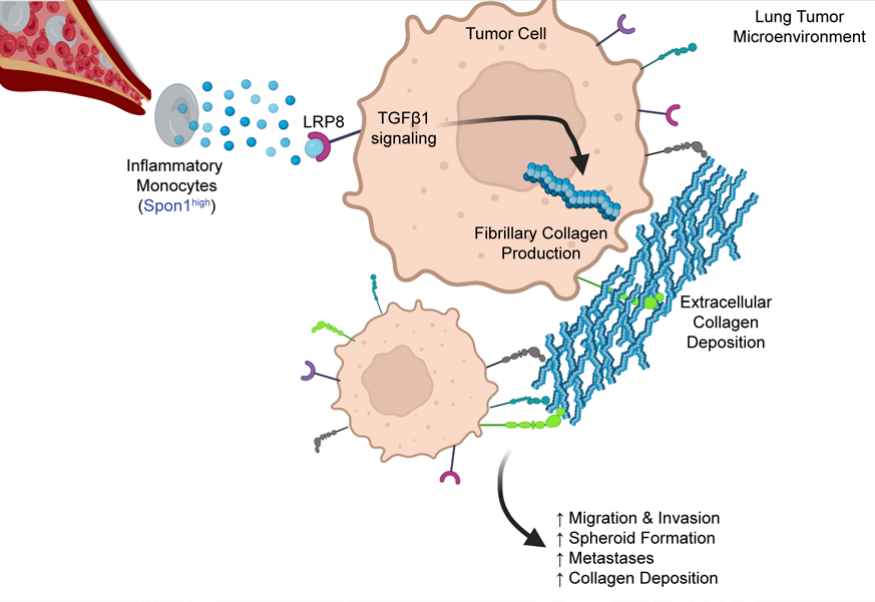
Schematic of Spon1^hi^ TIMs altering the LUSC TME. Spon1^hi^ TIMs are recruited to the TME and bind to their receptor, LRP8, located on cancer cells. Through TGF-β1 activation, they enhance upregulation of our Collagen Gene Signature, which includes several fibrillar collagens. This then leads to extracellular collagen deposition and increased protumorigenic phenotypes such as migration, invasion, spheroid formation, and metastases.

Next, we used tissue microarray (TMA) cores of patients with NSCLC to evaluate the relevance of the SPON1/LRP8/TGF-β1 axis in clinical samples. Consistent with our bioinformatic, in vitro, and in vivo findings, CCR2^+^ TIMs in patient samples expressed SPON1 in tumors (Figure 7A). While these SPON1^+^ TIMs were about 5 times more abundant in the stroma (PanCK^–^) as compared with cancer cell islets (PanCK^+^), the SPON1^+^ TIMs in the cancer cell islets were associated with collagen deposition in the tumor (Supplemental Figure 8). This suggests that juxtacrine interactions of SPON1^+^ TIMs with cancer cells may be sufficient to promote collagen production. NSCLC cells consistently expressed LRP8 (Figure 7B); however, we observed variable percentages of dual LRP8^+^ and pSMAD2^+^ staining, indicating a dynamic activation state of TGF-β1 in the cancer cells. Collagen was deposited right around the tumor islets (Figure 7C), providing evidence for TGF-β1–induced collagen deposition in the tumors. Also, the density of SPON1^+^ TIMs was modestly correlated with LRP8^+^ and pSMAD2^+^ cancer cells (*r* = 0.27, *P* < 0.0001) (Figure 7D), further suggesting an association between SPON1^+^ TIMs in the tumor and LRP8-associated TGF-β1 activation in cancer cells. SPON1^–^CCR2^+^ cells, however, did not show this association and were negatively correlated with LRP8^+^ and pSMAD2^+^ cells. Dual cancer LRP8^+^ and pSMAD2^+^ staining was also found to be positively correlated with HDM, albeit more modestly (*r* = 0.14, *P* = 0.008), further connecting LRP8-induced TGF-β1 activation with collagen deposition (Figure 7E). Finally, high levels of collagen deposition within resected NSCLC tumors were strongly associated with poor survival in patients with NSCLC (*P* = 0.0057) (Figure 7F). Taken together, our work demonstrates that SPON1^+^ TIMs promote collagen remodeling in the TME through an LRP8/TGF-β1 signaling axis (Figure 8), which results in prometastatic conditions for NSCLC progression.

## Discussion

NSCLC is among the most aggressive of all cancer types. The high mortality rates are related to patients presenting at advanced stages and the tumors typically developing resistance to conventional therapies (50). With the recent advances of ICIs, the 5-year overall survival for patients with NSCLC has improved from a previous ~5% rate to nearly 20% (51). In unselected patients with NSCLC, only about 20% of tumors respond to these immunotherapies; thus, it is crucial to develop new therapeutic options (52). To address this need, various combinatorial therapies are being tested with anti–PD-1 and –PD-L1 immunotherapies, such as VEGF inhibitors and CTLA4 blockade (53). The involvement of myeloid cells in NSCLC progression is of growing interest from both a biological and therapeutic perspective (54). Importantly, the major NSCLC subtypes (LUSC and LUAD) can become highly enriched with TIMs, which is associated with increased metastatic tumor biology and poor overall survival (25, 28).

Previous studies have found that genetic or pharmacologic inhibition of TIMs can decrease disease progression in several mouse models. One study evaluating breast cancer found that depleting tumor–derived CCL2 resulted in inhibition of metastases (55). Other groups have shown that use of *Ccr2*^–/–^ mice or CCR2 inhibitors in murine models also resulted in decreased disease progression in many cancer types (24, 56–59). In LUSC and LUAD models, blocking CCR2 to prevent IM recruitment resulted in reduced metastases (25, 60). While several murine models respond to pharmacological inhibition of inflammatory monocyte recruitment into the tumor, thus far, the same therapeutic effects have not been observed clinically. For example, a clinical trial with PF-04136309 (NCT02732938), a CCR2 inhibitor that targets TIMs, did not augment the efficacy of nab-paclitaxel plus gemcitabine in patients with metastatic pancreatic cancer (61). Another trial that tested Carlumab, an anti-CCL2 antibody, in combination with chemotherapy showed an overall survival rate of only 5%, despite observing decreases in free CCL2 (NCT01204996PF) (62). Despite a decrease in monocytes and M2-like TAMs, Axatilimab, which targets the CSF1R-CSF1 axis to block monocyte recruitment to the tumor, also showed no added response in combination with immunotherapy (NCT03238027) (62). One reason for the differences seen between responses in mouse models, as opposed to humans, may be due to ubiquitous targeting of multiple monocyte populations. The use of single-cell RNA-Seq highlights the heterogeneity of monocyte subsets within lung tumors in both humans and mice (63). Some monocyte populations, such as patrolling monocytes, which are recruited to the tumor through CX3CR1, have shown to promote antitumor immunity such as activating NK cells (59). Monocytes can produce molecules such as IL-12 and CXCL10 to reduce angiogenesis, trigger M1 macrophage and dendritic cell differentiation, recruit lymphocytes, and initiate tumoricidal functions through antibody-dependent cellular toxicity and TRAIL secretion (62, 64, 65). Furthermore, TIMs are a critical source of IFN-β, the production and signaling of which lead to T cell expansion (66). Since T cell expansion is a fundamental process for effective ICI activity, losing TIM-mediated T cell expansion may result in a lack of therapeutic effect in patients. Taken together, these divergent roles of monocytes as a whole, and TIMs in particular, create challenges in therapeutically targeting these myeloid populations. Furthermore, the pitfalls of inadequate specificity of monocyte targeting underscores the importance of studying the downstream effects of TIMs so that their negative effects on the TME can be blocked without tempering their potential contributions to antitumor immunity.

To identify insights into the downstream biologic effects of TIMs, we integrated genomics analyses from our NSCLC models and TCGA patient data. Using biological validation in in vitro and in vivo systems, we (a) determined specific molecules increased in TIMs and (b) identified a previously unappreciated mechanism by which TIMs exert their effects on tumor progression. We found that TIMs are significant producers of F-Spondin (SPON1), which has predominately been studied as a neuronal axon guidance molecule that is expressed in the developing nervous system (67–73). Interestingly, SPON1 has been shown to be upregulated in several cancers, including neuroblastoma and chondrosarcoma, and it has also been found to have a role in promoting neuroblastoma by leading high IL-6 expression through the MEKK/p38 MAPK/NF-κB–dependent pathway (74, 75). SPON1 has also been shown to play a role in cartilage metabolism in an osteoarthritis model, and SPON1-mediated cartilage degradation was found to be dependent on active TGF-β and PGE2 (76). This work also found that increased levels of tartrate-resistant acid phosphatase led to increased bone synthesis and turnover in *Spon1^–/–^* mice (77). SPON1 has also been found to increase osteosarcoma cell motility through an Fak/Src-dependent pathway (42). While these data support an emerging role of SPON1 in cancer progression, all studies thus far have focused on cancer-derived SPON1 and have not considered the role of SPON1 from the TME. To our knowledge, we are the first to reveal the role of TIMs as an important source of SPON1, which we demonstrate robustly promotes fibrillar collagen production via ApoER2-mediated (LRP8-mediated) TGF-β1 activation. Furthermore, either loss of *Spon1* in TIMs or genetic deletion of *Lrp8* expression on tumor cells was sufficient to significantly abrogate NSCLC metastases and collagen content within the TME. As supported by our TMA analyses, SPON1 TIM expression is coordinated with dual LRP8^+^ and pSMAD2^+^ cancer cell expression, which is also correlated with collagen deposition and poor prognosis.

CAFs are largely considered the major cellular producers of collagen in the TME (43, 78, 79). Collagens within the ECM have well-documented roles in promoting cancer progression — such as through promoting EMT, angiogenesis, and creating a protumoral immune TME — and recent studies demonstrate that type I collagen promotes tumor growth through mitochondrial biogenesis (80). TIM infiltration is associated with worse prognosis in several types of cancers (24, 56). In colorectal cancer (CRC), CAFs sorted from CCR2-deficient tumors showed aberrant collagen production such as reduced collagen I and XIV and increased collagen VI, identifying an interaction between TAMs/TIMs and CAFs (57). Work from Afik et al. also showed increases in type 1, 3, 6, and 14 collagen production by TIMs, and Afik et al. also observed *Spon1* as upregulated in TAMs (57), suggesting that monocyte-derived TAMs could remodel the ECM both intrinsically and through their regulation of CAFs. However, the mechanism by which collagen production is regulated by either TAMs or CAFs had remained to be elucidated. Importantly, our work uncovers an intercellular SPON1/LRP8/TGF-β axis that bridges the gap between SPON1^+^ TIMs and ECM remodeling, while also identifying tumor cells as a key producer of collagen.

Corroborating our findings in patients with NSCLC, another group recently found that an ECM-high set of LUSC tumors had a worse survival prognosis due to a profibrotic matrisome, which was also associated with increased invasiveness in LUSC (81). They also observed that high levels of fibrillar collagens was correlated with increased numbers of myofibroblasts in the tumors (81). Similar to our findings in *SPON1*^hi^ TCGA LUSC tumors, they also found that ECM-high tumors were highly enriched in EMT and TGF-β signaling signatures (81). CAFs have also been shown to produce several of the collagen genes we observed in the *SPON1*^hi^ LUSC tumors, such as *POSTN*, *COMP*, *GREM1*, and *THBS2* (48, 49, 82–84). However, there is emerging evidence showing support of cancer cell–derived collagen in the TME. For example, one group found that Hep3 cancer cells were able to produce type 3 collagen, and another group found matrix signatures highlighted in tumor cells through proteomic analyses of tumors (85, 86). The proteomic analysis also revealed upregulation of several types of collagens in both tumor cells and stromal cells (86). Building upon these previous findings, we identified a mechanistic link by which SPON1^+^ TIMs signal through LRP8^+^ cancer cells to activate TGF-β1, resulting in robust cancer cell production of fibrillar collagens. Interestingly, we also found that levels of LRP8 expression on CAFs were comparable with those of our cancer cell lines, and their ability to respond to SPON1 to produce collagens was also comparable. These intriguing findings suggest that cancer cells may model “CAF-mimicry,” such as when establishing and promoting a micrometastatic niche. Thus, while CAFs’ roles in collagen production are well established (87), this work reveals a previously unappreciated role of how SPON1^+^ TIMs can induce collagen deposition within the ECM via tumor cells.

This new SPON1/LRP8/TGF-β1 axis helps bridge the gap between the poor prognosis of TIM-high patients with collagen production. Moreover, as a receptor expressed on the cancer cells, LRP8 represents a therapeutic target for blocking TIM-mediated metastases without targeting the TIMs directly. Given the challenges thus far in developing effective myeloid targeting drugs in the clinic (88), which may be related to TIMs having both pro- and antitumoral functions, it’s possible that selective inhibition of key downstream pathways may prove a more effective therapeutic strategy.

## Methods

Supplemental Methods are available online with this article.

### Sex as a biological variable.

These studies included both male and female animals, although sex was largely not considered a biological variable, since lung cancer equally effects each sex.

### Cell lines and reagents.

LLC cells were obtained from ATCC, and parental KAL cells were provided by Yinling Hu (National Cancer Institute, Frederick, Maryland, USA). The LLC, parental KAL, and LN2E1 cell lines were cultured in DMEM (Thermo Fisher Scientific, 11995-065) supplemented with penicillin-streptomycin (Thermo Fisher Scientific, 15140-122) and 10% FBS (89). They were incubated in 0.25% Trypsin-EDTA (Thermo Fisher Scientific, 15050-065) for 5–10 minutes at 37°C for passaging. The LN4K1 (25) cell line was cultured in MEM (Thermo Fisher Scientific, 11095-080) supplemented with penicillin-streptomycin and 10% FBS. They were incubated in 0.25% Trypsin-EDTA for 10–15 minutes at 37°C for passaging. All cells were housed at 37°C in 5% CO_2_. For in vitro experiments, SB431542 (Selleckchem S1067) was used at 10 μM, and KRFK (MedChemExpress HY-P3970A) was used at 50 μM.

### Bioinformatics and statistics of RNA-Seq data.

Fastq files were inspected using FastQC. In particular, we looked for abnormal values of the sequence quality scores (https://www.bioinformatics.babraham.ac.uk/projects/fastqc/). Then, we performed adapter trimming on all available fastq files using BBDuk (https://jgi.doe.gov/data-and-tools/software-tools/bbtools/bb-tools-user-guide/bbduk-guide/), based on the adapters.fa file provided by BBMap (V37.99). This step was followed by short read alignment to the murine genome (MM10-UCSC) using BBMap (https://jgi.doe.gov/data-and-tools/software-tools/bbtools/bb-tools-user-guide/bbmap-guide/), with Illumina R1 and R2 files jointly used for paired-end alignment and in multithread modality (number of threads = 4) through a UNC-Chapel Hill UNIX-based cluster. The generated output (bam files) was sorted using samtools; later, these raw data were quantified using featureCounts of Subread (90, 91); for these computational steps, see also the family soft file available in Gene Expression Omnibus (GEO), accession no. GSE260524.

We modeled the gene expression and performed the statistical tests of intergroup differences using DESeq2. To leverage the multiple options offered by DESeq2 for distinct tasks (92), we tested the gene expression differences by running the DESeq function for each intergroup comparison. Fold changes (FCs) were calculated using normal-based shrunk values (normalized counts). This choice was suggested by our intention to position our statistical analysis close to the original framework proposed by Love et al. (92) and to produce results generally more conservative both with regards to untransformed data and to other shrinkage methods suggested by the DESeq2 authors at the time when we performed our research. In each comparison, we selected genes with an adjusted *P* < 0.0001 and |log_2_FC| > 1 after the aforementioned shrinkage versus 2 baselines. Then, we took the intersection among these sets of genes. Finally, to maximize the output clarity for data visualization, we regularized log–transformed (r-log–transformed) the expression values with the option “blind=FALSE” within the DESeq2 function r-log, following the authors’ guidelines.

The selected genes were alphabetically sorted, and their r-log–transformed gene expression values were visualized by heatmaps after being clustered. For this task, we used Cluster 3.0, limiting our processing to median-centering, without any further transformation or adjustment. For the clustering, we selected the hierarchical clustering option of average linkage based on the correlation similarity metric for genes (= matrix rows) only. Finally, these results were visualized using TreeView after setting the contrast value to 2.0 (93–95). Notably, the results shown in Figure 1B are based on 2 strains of mice (FVB and DBA2) from The Jackson Laboratory, so results could also be assessed for not being single-strain dependent. In the left panel of Figure 1B, we included genes that were differentially expressed both in the primary tumor and in the lymph nodes. These steps aimed at coupling the error mitigation provided by technical replicates (5 for each experimental condition and 3 for each baseline) (96) with the identification of consistent variations versus the baseline across tumor tissues (primary tumor and lymph node metastases for FVB mice and lung metastases for DBA2 mice).

### TCGA bioinformatic analyses.

mRNA gene expression of TCGA-LUSC was downloaded from gdac firehose (https://gdac.broadinstitute.org/). Patients were divided into 2 groups by *SPON1* gene expression. We used DESeq2 to identify DEGs between *SPON1*^hi^ (> median expression) and *SPON1*^lo^ (≤ median expression) groups (92).

Gene set analysis was done using 2 methods. We first calculated the significance of occurence in overlaps between DEGs and selected gene sets (msigDB: Hallmark, C5 and C2) using fisher exact test. The DEGs threshold used was adjusted *P* < 0.001, log_2_FC > 1.5 and baseMean > 10.

We also used the GSEA method to calculate enrichment scores (97). We ranked all genes by –log_10_ *P* value × log_2_FC and calculated their enrichment scores. SPON1 survival analysis was conducted using the Oncolnc TCGA data portal using the search term “SPON1” in the “LUSC” data set, with the lower and upper percentiles both being set to 50% (98). The Kaplan-Meier survival curve was generated using Graphpad Prism.

### Single cell GEO 10X analysis.

Single cell 10X data was downloaded from GEO data sets suggested as source material; 2 mouse liver cancer samples enriched for fibroblasts from GSE160541 (“Tumor-suppressive and tumor-promoting roles of CAF-released mediators in desmoplastic liver metastasis”; GSM4874984 and GSM4874983). The samples were analyzed using the R v4.4.1 package Seurat, v4.2.0 (99). Samples were individually filtered to drop genes found in < 3 cells and then to drop cells based on per-sample, manually curated thresholds for genes/cell detected and the percentage of mitochondrial genes. Using Seurat’s defaults, filtered results for each sample were log normalized and scaled. Then, principal component analysis (PCA) was performed on the most variable 2,000 genes to reduce the number of dimensions, and the PCA-reduced cells were clustered. Uniform Manifold Approximation and Projection (UMAP) (100) was applied to the clustered results for cluster visualization as a 2D image. The R Bioconductor package SingleR (101) was used to assign a major cell type to each cell using the ImmGen reference (102) accessed via the R Biconductor celldex package. Cluster and cell type–specific gene expression was explored for Spon1 and Lrp8.

Twenty-six human single cell 10X data sets from GSE127465 (36) were downloaded from GEO (103). The samples were analyzed using Seurat v4.4.1 (99) under R v4.2 (104). Samples were merged (not integrated), and cells with less than 3 features, a total expression < 300 counts, or a mitochondrial expression percentage > 20% were dropped. Features present in fewer than 3 cells were also dropped. Using Seurat’s defaults, filtered results were log normalized and scaled. PCA was performed on the most variable 2,000 genes to reduce the number of dimensions, and the PCA-reduced cells were clustered. UMAP (105) was applied to the clustered results for cluster visualization as a 2D image. The R Bioconductor package SingleR v2.2.0 (101) was used to assign a minor cell type to each cell using the ZilionisLungData data set from the R package scRNAseq v2.14.0 (106). For comparison, the original cell type assignment from GSE127465 (36) was downloaded. Spon1 expression was explored and aggregated normalized expression was reported.

### Spheroid assays and quantification.

Cells were embedded in Matrigel Matrix (Corning, Phenol Red Free, 356237) by resuspending 5 × 10^3^ cells in a 50 μL volume. Either complete media or complete media with 5 μg/mL recombinant mouse SPON1 (R&D Systems, 7950-SP-050) was added after the matrix cell suspension solidified by placing at 37°C for 15–30 minutes. For coculture experiments using IMs after monocyte isolation, 1 × 10^5^ cells were added to each spheroid mound with complete media. Freshly isolated IMs were added sequentially for 3 days. Cells were imaged between days 3 and 5, and samples were collected in RNA lysis buffer and analyzed by quantitative PCR (qPCR). Spheroid experiments were repeated 3 times, with biological replicates ranging from 50 to 200 spheroids.

### qPCR.

For mRNA quantification, total RNA was extracted from cells using the Quick RNA MicroPrep Zymo Research Kit (Genesee Scientific). cDNA was synthesized using standardized RNA using an iScript cDNA synthesis Kit (Bio-Rad) as per the manufacturer’s instructions. Specific primers for genes are listed in Supplemental Table 5. PCR was done with reverse-transcribed RNA, 1 μL each of 20 μM forward and reverse primers, and 2 × PowerUp SYBR Green Master Mix in a total volume of 25 μL. Each cycle consisted of 15 seconds of denaturation at 95°C and 1 minute of annealing and extension at 60°C (40 cycles). Reactions were run in triplicate or quadruplicate.

### CRISPRMax transfection.

Opti-MEM, Cas9 2NLS Nuclease, guide RNAs (Synthego) to LRP8, and Lipofectamine CRISPRMAX Cas9 Plus Transfection Reagent were added to a diluted CRISPRMAX reagent and incubated for 5 minutes to form complexes. gRNA sequences are listed in Supplemental Table 6. The CRISPRMax transfection was then added to either LN2E1 or LLC cells for 2–3 days at 37°C in 5% CO_2_. KO was confirmed by qPCR and Western blot.

### Animals, in vivo models, and tissue processing.

Adult FVBn or C57BL/6 mice were purchased from The Jackson Laboratory. Spon1^–/–^ mice were provided by Steven Abramson (NYU Langone Health). Male and female animals were between 8 and 15 weeks at the time of injection. For all animal experiments, cells were trypsinized, washed, and resuspended in HBSS (Thermo Fisher Scientific) prior to injection. Orthotopic injections were performed by an intrapulmonary technique after anesthesia with ketamine (80 mg/kg) + xylazine (8 mg/kg) + acepromazine (1 mg/kg) and placed in the right lateral recumbency. Following fur removal and sterile skin preparation, an incision parallel to the rib cage between ribs 10 and 11 was made to visualize the lung through the intact thoracic pleura. A 1 mL tuberculin syringe with a 30 gauge needle was used to inject the cell suspension directly into the lung parenchyma at the left lateral dorsal axillary line. After injection, the skin incision was closed using surgery clips, and the mice were turned on the left lateral recumbency and observed until fully recovered. Luciferase-labeled tumor progression was monitored using an IVIS Lumina optical imaging system and Nano-Glo Luciferase Assay substrate (Promega), as per the manufacturer’s instructions. For the adoptive transfer, IMs were isolated from BM of C57BL/6 mice from The Jackson Laboratory or Spon1^–/–^ mice, and cells were infused (1 × 10^5^ cells in 100 μL HBSS) on days 6, 8, 11 and 12, in their respective groups. On day 13, mice were sacrificed. In all experiments, 3–10 mice per group were used. For the T cell–depletion study, isotype (BioXCell, BE0086) and anti-CD8 (BioXCell, BE0061) were used at 400 μg/mouse twice per week. Once mice in any group became moribund, they were all sacrificed and necropsied, and tumors were harvested. Tumor weights and number, and the location of lymphatic and distant metastases, were recorded. Tissues used for IHC analysis were fixed in 10% neutral buffered formalin and embedded in paraffin.

### Monocyte isolation.

For monocyte isolation experiments, blood, BM, and tumors were collected for either monocyte isolation through magnetic separation (Stemcell Technology, 19861) and/or FACS. Lung tissues were washed and mechanically minced using a sterile scalpel in low-glucose DMEM and digestion media (1 mL collagenase at 2 mg/mL, and 15 μL DNase at 1 mg/mL). Tissue was digested into a single cell suspension by light shaking in digestion media for 30 minutes at 37°C and was then filtered through a 40 μM cell strainer, pelleted, treated with ACK lysis buffer at room temperature for 2 minutes, and then pelleted again. Cells were resuspend in FACS buffer (0.5% BSA and 2 mM EDTA in PBS) at a concentration of 1 × 10^6^ cells/100 μL. Samples were incubated with the following antibodies: CD45 (APC-conjugated, 103112, BioLegend), CD11b (PE/Cy5 conjugated, 101210, BioLegend), Ly6C (PE/Cy7-conjugated, 128018, BioLegend), Ly6G (PE-conjugated, 127608, BioLegend), Siglec-F (BV421-conjugated, 562681, BD Bioscience), F4/80 (FITC-conjugated, 123108, BioLegend), and LIVE/DEAD Fixable Violet Dead Cell Stain (L34963, Thermo Fisher Scientific). Approximately 0.2 μg of antibody was used for every 1 × 10^6^ cells. Cells were incubated with antibody for 40 minutes on ice, in the dark. Cells were then washed 2 times with PBS and taken for FACS on a FACSAriaII. The collected data were analyzed using FCSExpress. When performing FACS on lung tissues, Siglec-F^+^ cells were included with dead cells to remove alveolar macrophages and/or eosinophils. IMs were identified as CD45^+^CD11b^+^Ly6C^hi^Ly6G^–^ cells (Supplemental Figure 9). Alveolar macrophages were isolated from healthy lungs of C57BL/6 mice using the same isolation procedure above and the following FACS antibodies: CD45 (BV605-conjugated, 567459, BD Bioscience), CD11b (PE/Cy5 conjugated, 101210, BioLegend), Siglec-F (BB515-conjugated, 564514, BioLegend), and LIVE/DEAD Fixable Near-IR Dead Cell Stain (L34975, Thermo Fisher Scientific). Alveolar Macs were identified as CD45^+^CD11b^–^Siglec-F^+^.

### Picrosirius red staining and analysis.

Picrosirius red staining was performed on formalin fixed, paraffin embedded tumor sections using a commercially available Picrosirius Red Stain Kit (Polysciences, 24901-500). Slides were deparaffinized and hydrated to deionized water before being placed in phosphomolybdic acid for 2 minutes. Slides were then thoroughly rinsed in water before being placed in the Picrosirius red F3BA stain for 60 minutes. They were then placed straight into 0.1N Hydrochloric Acid for 2 minutes and washed in 70% ethanol for 45 seconds before they were dehydrated, cleared in xylene, and then mounted using organic mounting media (Permount).

Stained slides were imaged using a Leica DMi8 inverted microscope for bright-field microscopy. Image processing was done using a TWOMBLI (44) macro to quantify extracellular positive staining in the FIJI software.

### CAFs from mouse lungs.

For lung CAF isolation, lung tumors from LSL-KRAS^G12D/+^ p53^fl/fl^ Lkb1^fl/fl^ mutant mice were minced and subjected to a 90-minute incubation at 37°C in CAF dissociation buffer composed of 2 mg/mL of collagenase type I (Worthington), 1 mg/mL of DNase (Worthington), and 2.5 units/mL of neural protease (Worthington). Subsequently, the digested tissues were transformed into a single-cell suspension by passing through a 100 μM cell strainer. The upper layer of hydrolyzed fat was discarded. Endothelial cells and hematopoietic cells were removed using CD31 or CD45 magnetic beads as previously described (107). The resultant fraction was then centrifuged at 400*g* for 5 minutes, washed 2× with HBSS, and incubated on plastic dishes for 30 minutes. Following this incubation period, nonadherent cells were thoroughly washed away, and the adhesive cells were cultured in high-glucose DMEM supplemented with 20% FBS and 10 ng/mL basic FGF. Non-CAF cells were eliminated from the culture to generate a pure CAF population. Upon attaining homogeneous CAF cultures, cells were detached from the plate using Accutase and serially passaged no more than 10 times.

### Statistics.

Between 3 and 10 mice were assigned per treatment group; this sample size gave approximately 80% power to detect a 50% reduction in tumor weight with a 95% CI. Results for each group were compared using 2-tailed Student’s *t* test (for comparisons of 2 groups) and 1-way ANOVA (for multiple-group comparisons). The multiple-hypothesis testing correction of these statistical results was made using the FDR. A *P* value less than 0.05 was deemed statistically significant. All statistical tests for in vitro and in vivo experiments were performed using GraphPad Prism 7 (GraphPad Software).

### Study approval.

These animals were cared for according to guidelines set forth by the American Association for Accreditation of Laboratory Animal Care and the Public Health Service policy on Human Care and Use of Laboratory Animals. All mouse studies were approved and supervised by the University of North Carolina Institutional Animal Care and Use Committee.

### Data availability.

Bulk RNA-Seq data have been deposited and are accessible in the NCBI GEO database at accession no. GSE260524. Values for all data points in graphs are reported in the Supporting Data Values file.

## Author contributions

Conceptualization was contributed by NS and CVP; methodology was contributed by NS, LE, KMW, ATW, AP, MPZ, ACC, GY, ALA, and AD; investigation was contributed by DJK, NS, KMW, LE, SS, YST, AP, SRJ, ALA, ACD, and CVP; formal data analysis and visualization was contributed by NS, KMW, LE, SS, YST, AP, SRJ, and CVP; funding acquisition was contributed by CVP and NS; resources were contributed by CVP; writing of the original draft was contributed by NS and CVP; review and editing were contributed by all authors.

## Supplementary Material

Supplemental data

Unedited blot and gel images

Supplemental tables 1-6

Supporting data values

## Version 1 05/08/2024

Electronic publication
