# Supplementary material for: Spon1+ inflammatory monocytes promote collagen remodeling and lung cancer metastasis through lipoprotein receptor 8 signaling
Source: JCI Insight. 2024 May 8;9(9):e168792. doi: 10.1172/jci.insight.168792 (PMC11141919; doi:10.1172/jci.insight.168792)
Supplement: Supplemental data [file jciinsight-9-168792-s159.pdf]

Title: Spon1+ Inflammatory Monocytes Promote Collagen Remodeling and Lung Cancer Metastasis through Lipoprotein Receptor 8 Signaling

Authors: Kristina M. Whately, Nisitha Sengottuvel, Lincy Edatt, Sonal Srivastava, Allison T. Woods, Yihsuan S. Tsai, Alessandro Porrello, Matthew P. Zimmerman, Aaron C. Chack, Stuart R. Jefferys, Gabriella Yacovone, Dae Joong Kim, Andrew C. Dudley, Antonio L. Amelio, Chad V. Pecot

Supplemental Methods:

Immunostaining of TMA cores

Tissue microarray (TMA) samples for lung squamous cell carcinoma and lung adenocarcinoma cancers were obtained and prepared following Institutional Review Board approval for UNC Chapel Hill. Triple immunofluorescence (IF) was performed on paraffin-embedded TMAs that were sectioned at 4 microns onto positive charged slides. Tissue sections were stained with or labeled for antigens using two different triple stain assays. The first multiplex combination was with pSMAD2 (44-244G, Invitrogen), LRP8 (HPA073031, Sigma Aldrich), and pan Cytokeratin (NCL-L-AE1/AE3-601, Leica). The second triplex combination included SPON1 (Genetex, GTX51796), CCR2 (Abcam, ab176396), and pan Cytokeratin (NCL-L-AE1/AE3-601, Leica). These IF assays were carried out on the Bond Rx fully automated slide staining system (Leica Biosystems) using the Bond Research Detection kit (DS9455). Slides were deparaffinized in Leica Bond Dewax solution (AR9222), hydrated in Bond Wash solution (AR9590) and sequentially stained for either a triplex stain with pSMAD2, LRP8, and pan Cytokeratin or SPON1, CCR2, and pan Cytokeratin. Briefly, antigen retrieval was accomplished using Bond-epitope retrieval solution 1 pH 6.0 (AR9961). After pretreatment, tissues were blocked, and primary antibodies were diluted as follows: pSMAD2 at 1:3000, LRP8 at 1:100, pan Cytokeratin at 1:300 or with SPON1 at 1:300, CCR2 at 1:500, and pan Cytokeratin at 1:300. Ready-to use secondary antibodies Novolink Post Primary and/or Novolink Polymer (RE7260-CE, Leica) were used followed by either TSA Cy5 (SAT705A001EA, Akoya Biosciences), TSA Cy3 (SAT704A001EA, Akoya Biosciences) or Alexa Fluor™ 488 Tyramide Reagent (B40953, ThermoFisher Scientific) to visualize the target of interest. Nuclei were stained with Hoechst 33258 (Invitrogen). The stained slides were mounted with ProLong Gold antifade reagent (P36930, Life Technologies,). Positive and negative controls (no primary antibody) were included in this run.

Slides were digitalized using the Aperio ScanScope FL (Aperio Technologies Inc). The digital images were captured in each channel by 20x objective (0.468 µm/pixel resolution) using line-scan camera technology (U.S. Patent 6,711,283). The adjacent 1 mm stripes captured across the entire slide were aligned into a contiguous digital image by an image composer.

Sirius Red of TMA cores:

Paraffin embedded TMAs were sectioned at 4 microns onto positively charged slides. To proceed with histological staining, samples were first baked at 60 degrees Celsius for 60 minutes minimum and were then deparaffinized in xylene and hydrated with graded ethanols before continuing with the stain of interest. To detect collagen in tissues, the Sirius Red stain was used (24901-500, PolySciences). After the staining, slides were then dehydrated and coverslipped with Cytoseal 60 (8310-4, Thermo Fisher Scientific). Stained slides were digitally imaged in the Aperio AT2 (Leica Biosystems) using 20x objective.

ELISA

Murine SPON1 protein levels were quantified by ELISA using the Mouse Spondin-1(SPON1) ELISA kit (Biorbyt orb408655) according to the manufacturer's protocol. To assess secretion of SPON1 in vitro, LLC spheroids were seeded at a density of 50,000 cells per well in 500uL of media in 24-well plates. Supernatant was collected 5 days later and stored at −80 °C. For analysis of plasma SPON1 levels, blood (approximately 800 µL per mouse) from 3 to 5 mice per group was obtained at sacrifice via heart stick. Blood was collected into SST Blood Collection Tubes. Tubes were centrifuged at 10,000g for at room temperature for 10 min, then plasma was collected and stored at −80 °C until assay. Samples were assayed in triplicate and data represents the mean concentration.

Immunostaining

Formalin preserved tumor tissue was paraffin embedded and sectioned into slides at 5µm thickness. Slides were deparaffinized and rehydrated before undergoing antigen retrieval (Vector Lab Cat#3300) for 20 minutes. Then endogenous peroxidase was quenched using 3% hydrogen peroxide followed by avidin and biotin blocking (Vector Lab Cat#SP-2001). For pSMAD2 staining, slides were blocked with 10% goat serum for 1-2 hours at room temperature before incubation with primary rabbit polyclonal pSMAD2 (1:500 dilution, Invitrogen 44244G) in 3% goat serum overnight. Washed slides underwent secondary antibody treatment with biotinylated anti-rabbit IgG (Biocare Medical cat#GR608H) for 30 minutes. All slides were then incubated with Avidin-Biotin complexes for 30 minutes and washed before being developed in DAB solution (Impact DAB from Vector lab#SK-4105) and counterstained in Hematoxylin (Sigma-Aldrich #GHS316). IHC stained slides were digitally imaged in the Aperio ScanScope XT (Leica) using 20x objective and analyzed using cell profiler to count positively stained cells.

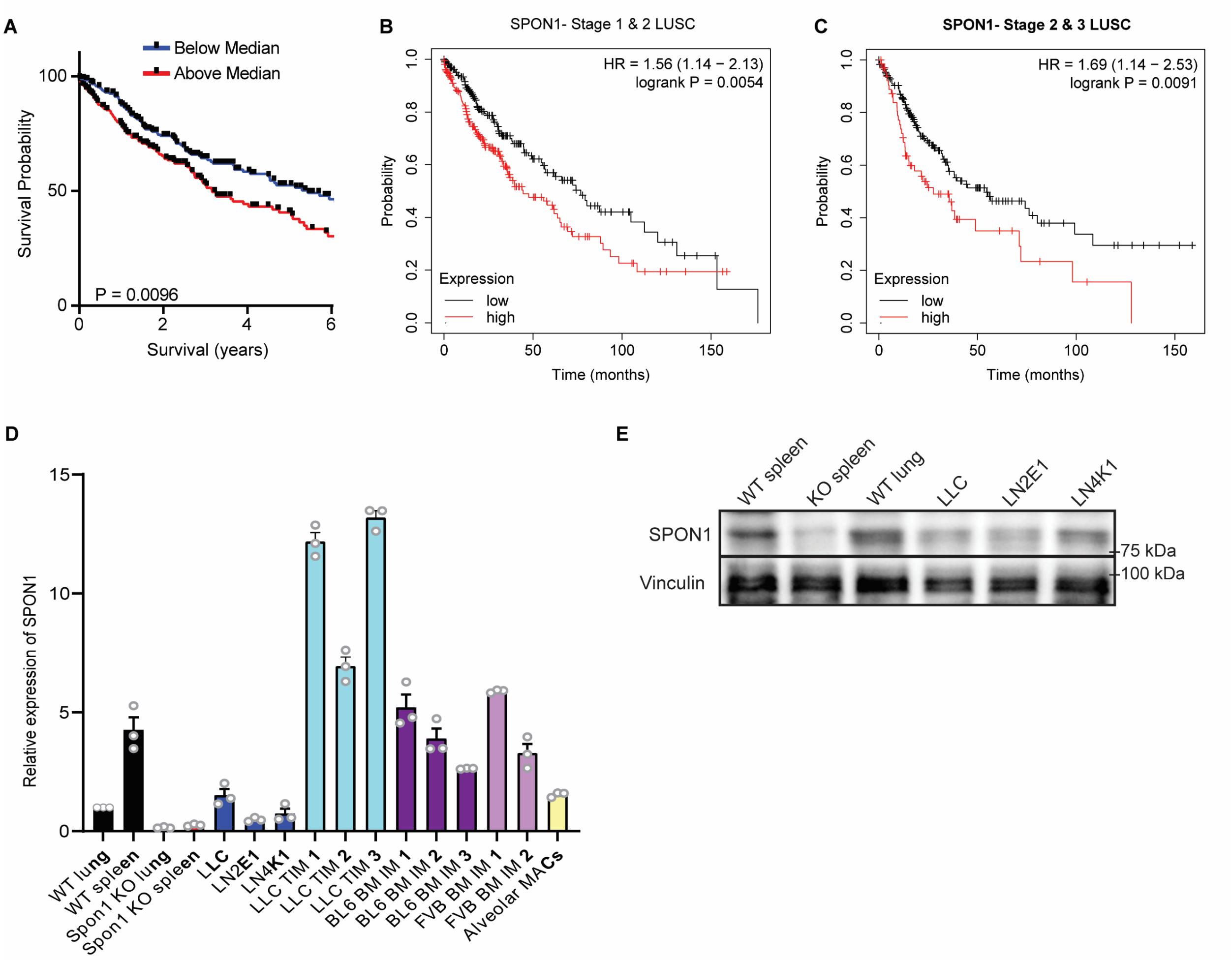

Supplemental Figure 1. (A) Survival differences between patients expressing high and low levels of Spon1 (n=244/group, p=0.0096) using a TCGA LUSC data set with a 50% upper and lower limit. (B) Survival differences between Stage 1 and 2 and (C) Stage 2 and 3 LUSC patients expressing high and low Spon1 levels using KMplot RNAseq datasets. (D) Spon1 gene expression of WT and Spon1$^{-/-}$ mouse organs, LUSC cell lines, LLC isolated TIMs, healthy BM IMs, and alveolar Macs normalized to WT mouse lung. (E) Protein expression of Spon1 and vinculin for lung cancer cell lines and mouse organ controls. Data are shown as the mean ± SEM incorporating biological and technical replicate samples.

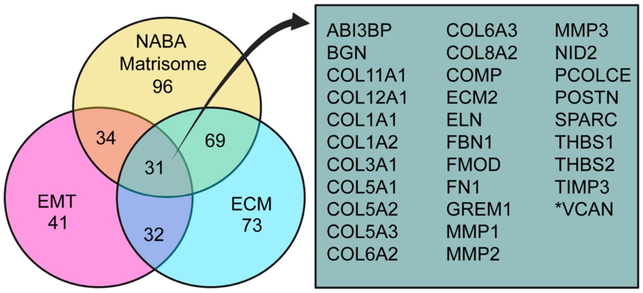

Supplemental Figure 2. Top hits in each gene-set show a collection of 31 overlapping genes.

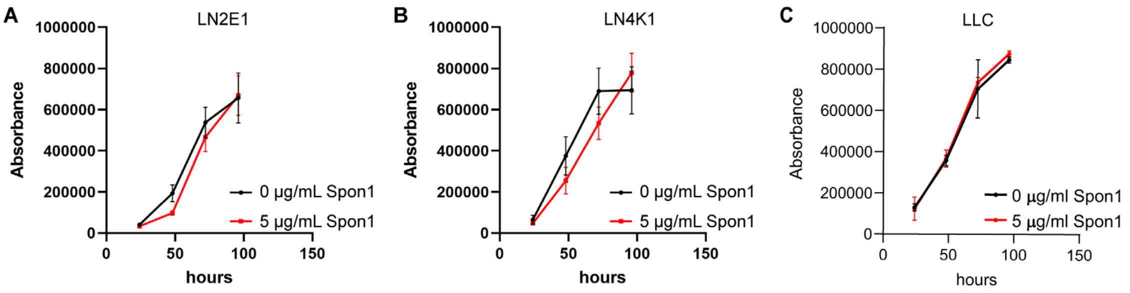

Supplemental Figure 3. Proliferation data shown with 2,000 of either (A) LN2E1, (B) LN4K1 or (C) LLC cells plated in the absence or presence of recombinant murine SPON1 (5µg/mL). Alamar blue readouts were conducted every 24 hours for 4 days and showed no differences in proliferation. Data are shown as the mean ± SEM incorporating biological and technical replicate samples.

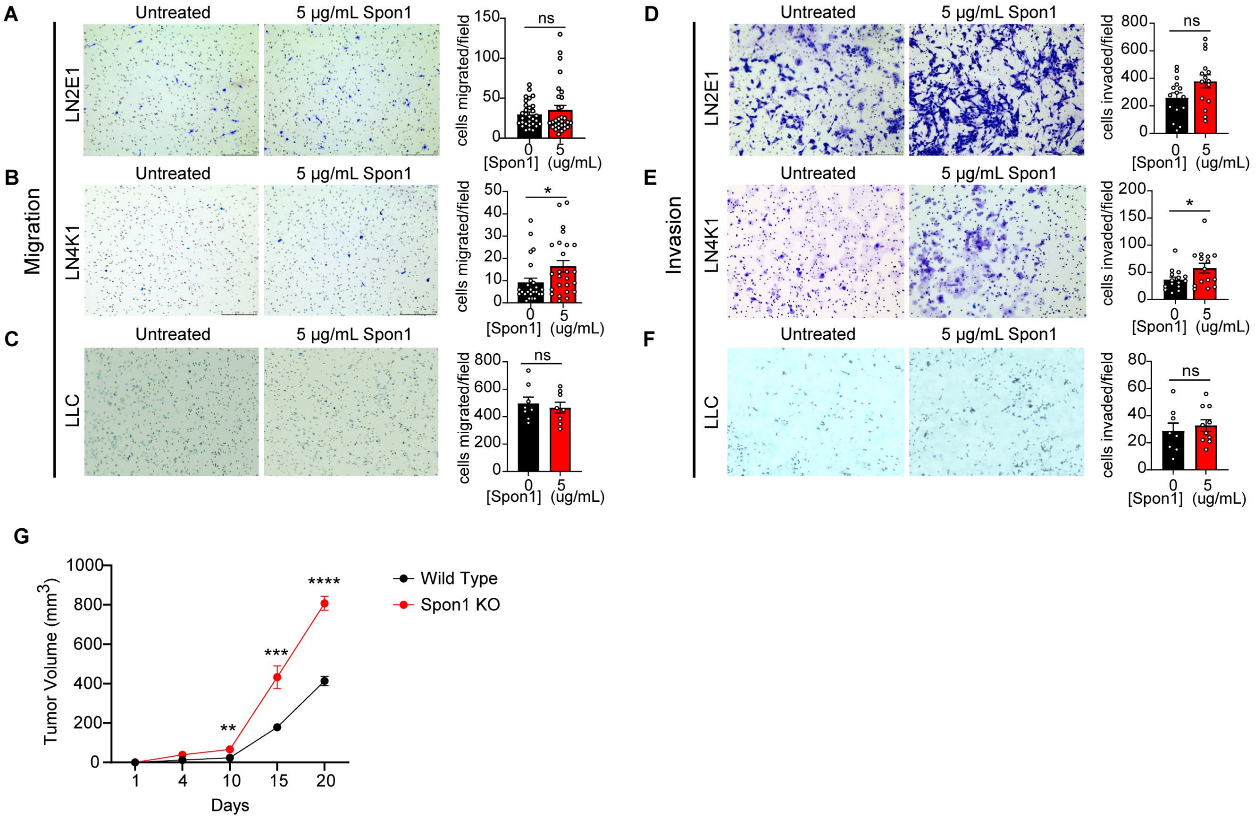

Supplemental Figure 4. To measure the ability of cells to migrate in the presence of a chemical gradient of recombinant mSPON1, an 8 µm transwell (Fisher 122-911) was used in a 24 well plate to create a barrier between the upper and lower chambers. Cells were placed in the upper chamber and mSPON1 (5µg/mL) + 5% FBS was placed in the lower chamber. Control wells contained 5% FBS only. Results shown for (A) LN2E1, (B) LN4K1, and (C) LLC cell lines with and without recombinant murine SPON1 treatment. Chambers were stained with the Hema 3 kit after 6 hours, mounted, imaged, and then analyzed using Cell Profiler. Invasion assays were done using a similar procedure with BioCoat Matrigel Invasion Chambers (Corning 354480) and an 18–22-hour incubation for (D) LN2E1, (E) LN4K1, and (F) LLC cell lines. (G) Subcutaneous LLC tumor growth in WT and Spon1$^{-/-}$ mice. (****) = p<0.001, (***) = p<0.001, (**) = p<0.01, (*) = p<0.05. Data are shown as the mean ± SEM incorporating biological and technical replicate samples. Two-tailed Student's t test for 2-group comparisons.

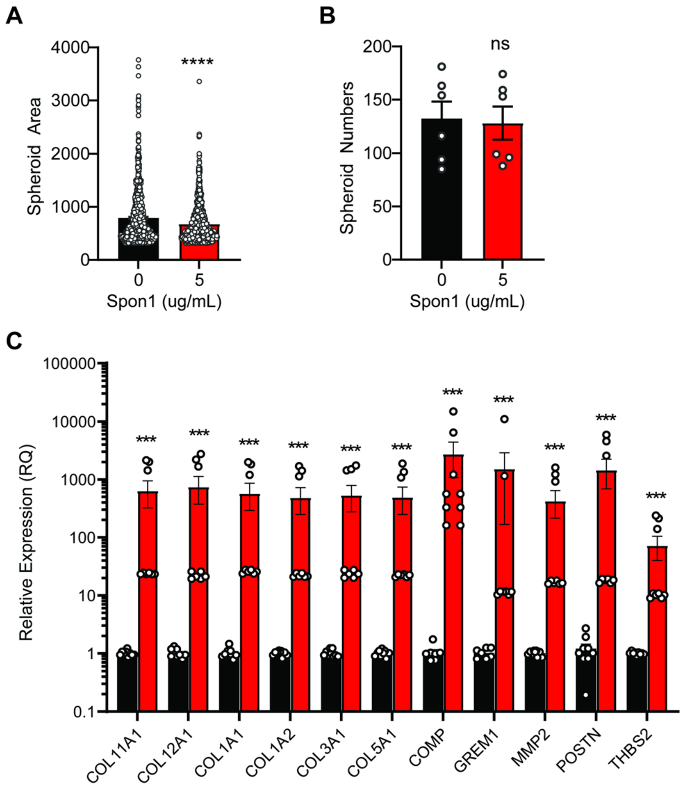

Supplemental Figure 5. A) Spheroid area and B) numbers for LN4K1. C) Collagen gene expression with and without recombinant murine Spon1 treatment (5μg/mL) for LN4K1 treated spheroids. (****) = p<0.0001, (***) = p<0.001. Data are shown as the mean ± SEM incorporating biological and technical replicate samples. Two-tailed Student's t test for 2-group comparisons.

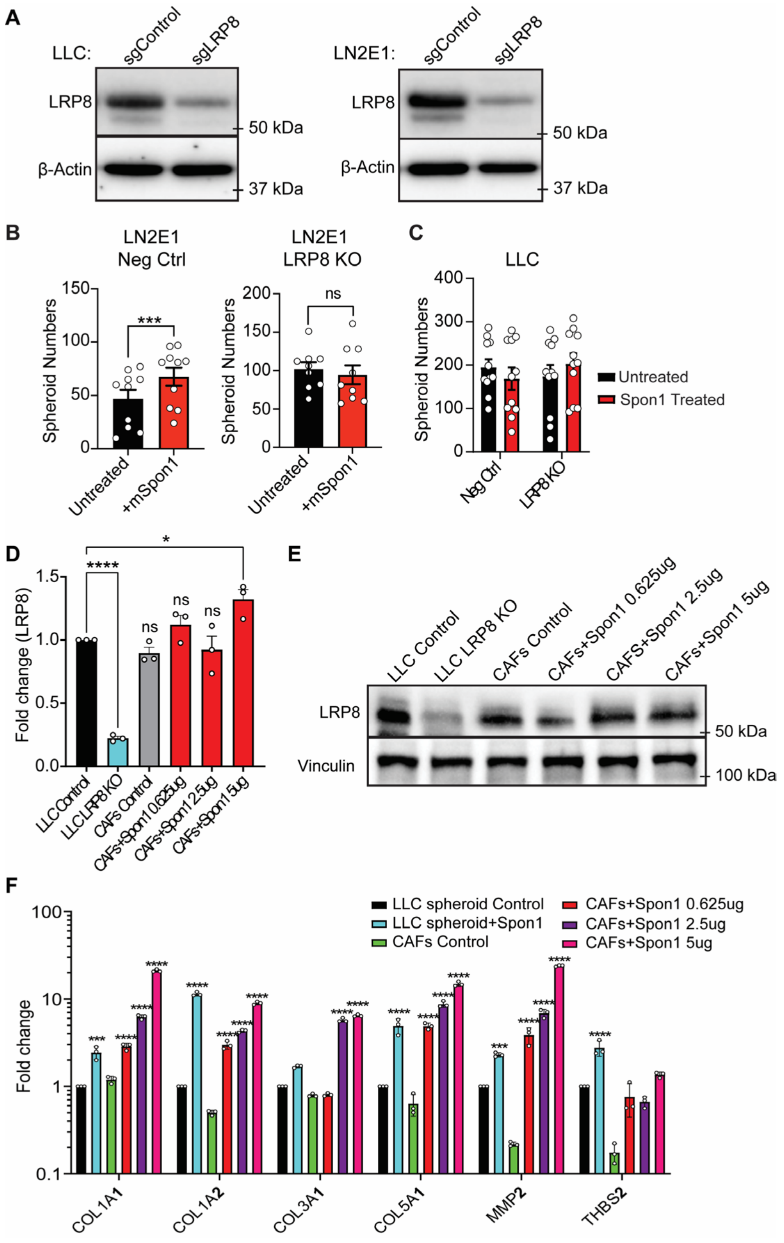

Supplemental Figure 6. A) Western blot confirmation for LRP8 KO in both LLC and LN2E1 cells. Spheroid formation phenotype for B) LN2E1 and C) LLC with and without recombinant murine Spon1 treatment (5 μg/mL) in negative controls and LRP8 KO cells. D) qPCR of Spon1 expression in CAFs untreated and treated with Spon1 compared to LLC cells. E) Protein expression of LRP8 and vinculin for CAFs untreated and treated with Spon1 compared to LLC cells. F) qPCR of collagen genes in CAFs untreated and treated with Spon1 compared to LLC spheroids. (****) = p<0.001, (***) = p<0.001, (*) = p<0.05. Data are shown as the mean ± SEM incorporating biological and technical replicate samples. Two-tailed Student's t test for 2-group comparisons; 1-way ANOVA test for multiple comparisons.

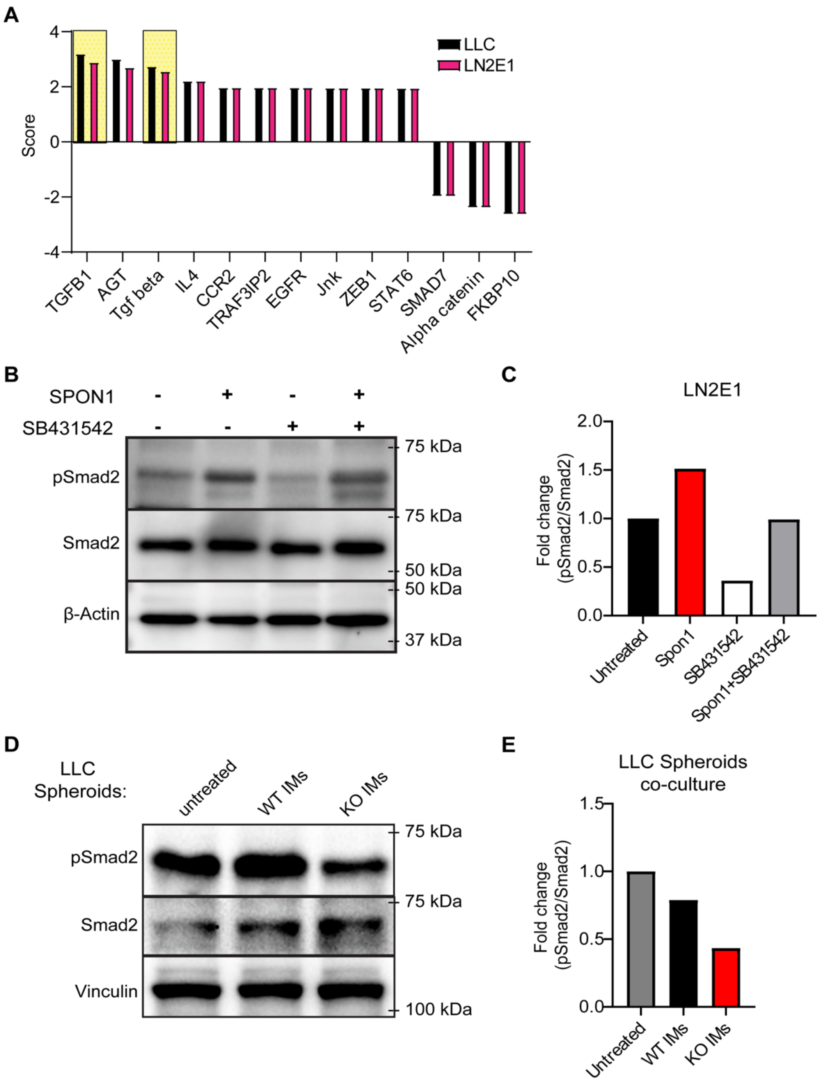

Supplemental Figure 7. A) Ingenuity Pathway Analysis Scores of common upstream regulators shown to drive collagen gene expression. TGFβ family members highlighted in yellow. B) Protein expression of phospho-smad2, total smad2 and actin from LN2E1 spheroids with or without recombinant Spon1 or 10uM SB431542. C) Quantification of phospho-smad2 to total smad2 from LN2E1 spheroids. D) Protein expression of phospho-smad2, total smad2 and actin from LLC spheroids with or without co-culture of WT or Spon1$^{-/-}$ IMs. E) Quantification of phospho-smad2 to total smad2 from LLC spheroids co-culture.

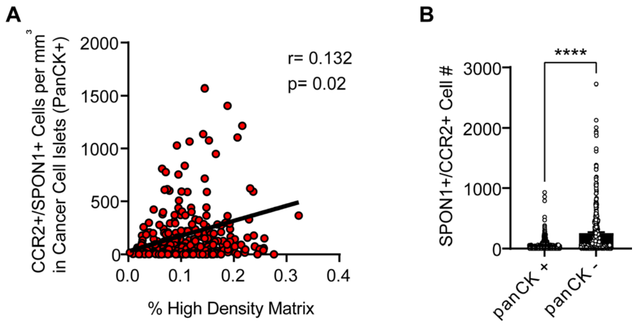

Supplemental Figure 8. NSCLC tissue microarray (TMA) data showing A) two-sided Pearson correlation between cells expressing both CCR2 and SPON1 in cancer cell islets (demarcated by positive panCK staining) with high density matrix of sirius red staining ($p=0.02$, $r=0.132$), and B) number of dual CCR2+/Spon1+ cells in PanCK positive vs PanCK negative regions. (****) = $p<0.0001$. Data are shown as the mean ± SEM incorporating biological and technical replicate samples.

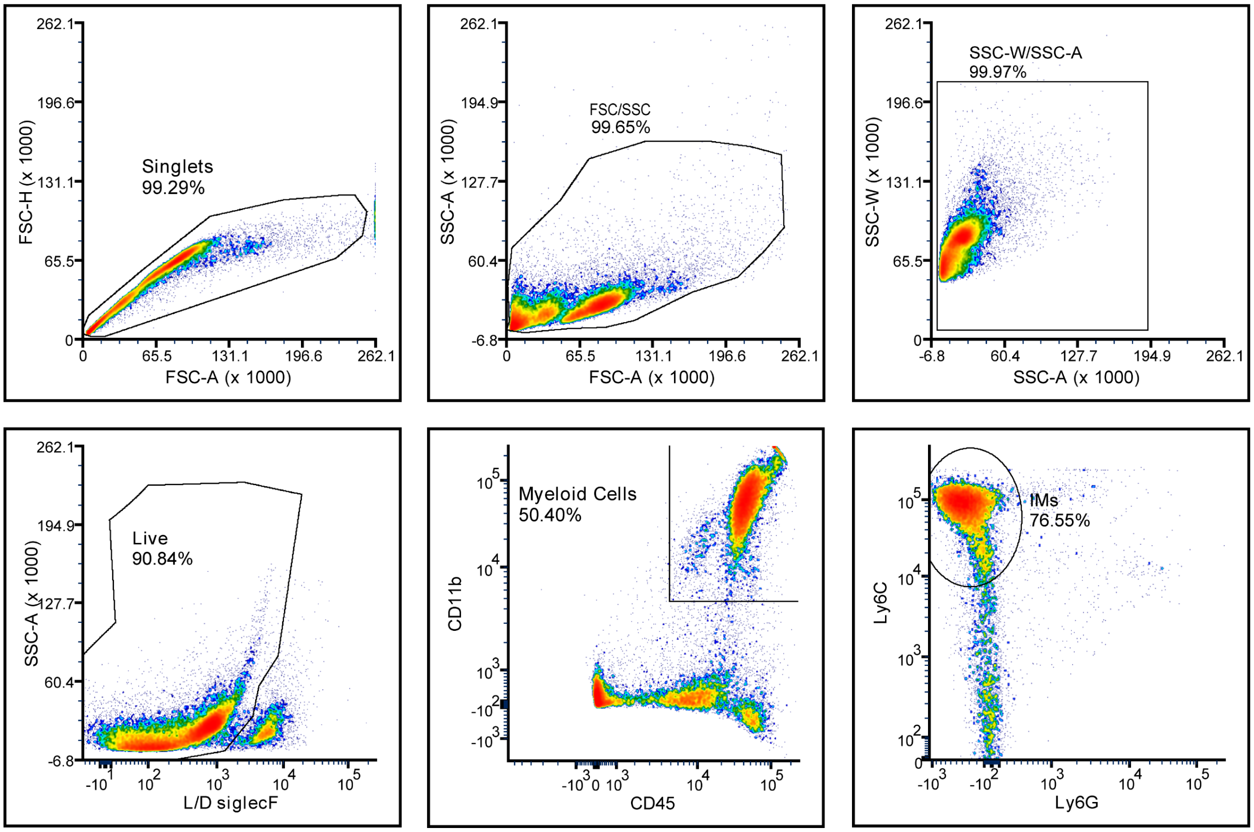

Supplemental Figure 9. Sample gating strategy for sorting of inflammatory monocytes from tumor samples and healthy bone-marrow. Sample gating shown on a control spleen that had been purified with the StemCell Monocyte Isolation kit. IMs were gated on Live SiglecF- cells as CD45+/CD11b+/Ly6G-/Ly6C^{High}.
