# Supplementary material for: Spon1+ inflammatory monocytes promote collagen remodeling and lung cancer metastasis through lipoprotein receptor 8 signaling
Source: JCI Insight. 2024 May 8;9(9):e168792. doi: 10.1172/jci.insight.168792 (PMC11141919; doi:10.1172/jci.insight.168792)

Full unedited blot for Supplementary Figure 1E:

SPON1:

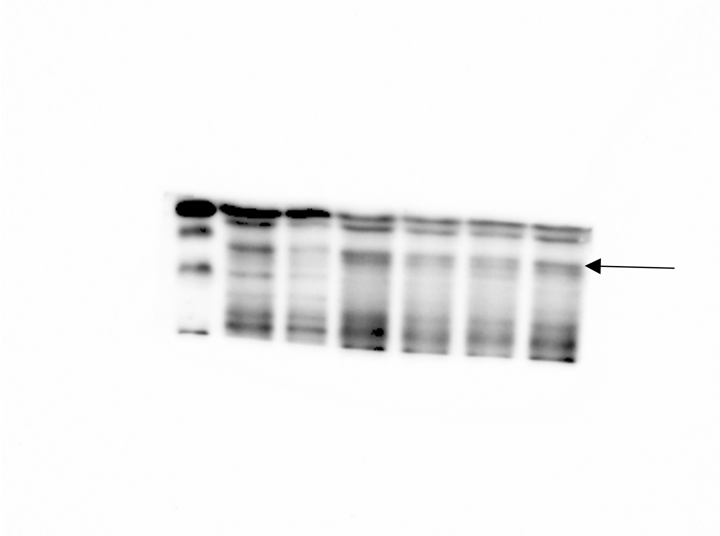

VINCULIN:

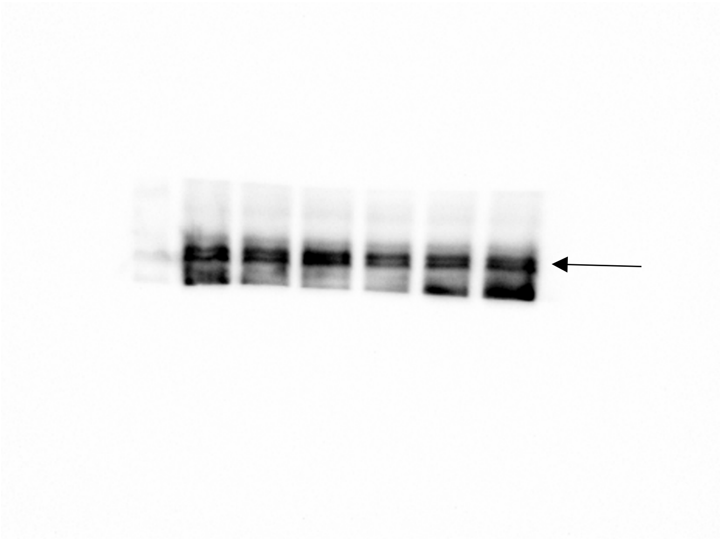

Full unedited blot for Supplementary Figure 6A:

LLC LRP8:

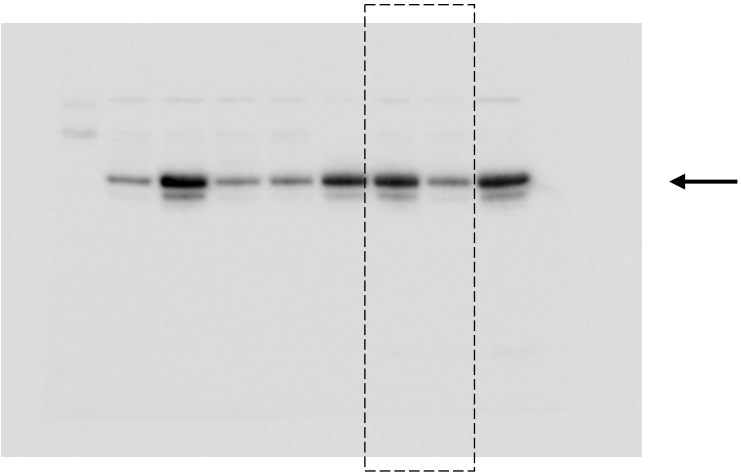

LLC β-ACTIN:

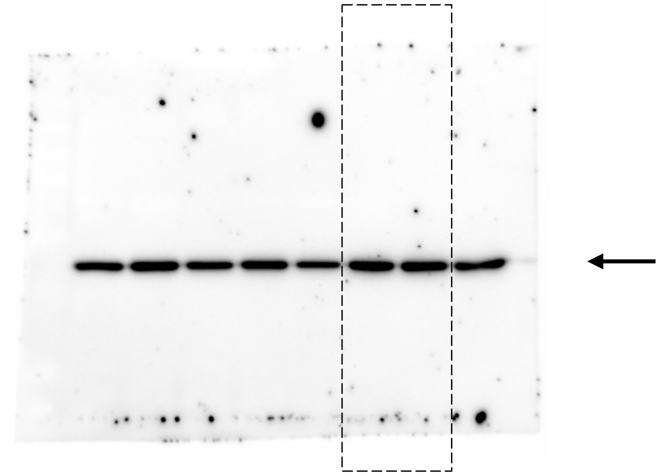

LN2E1 LRP8:

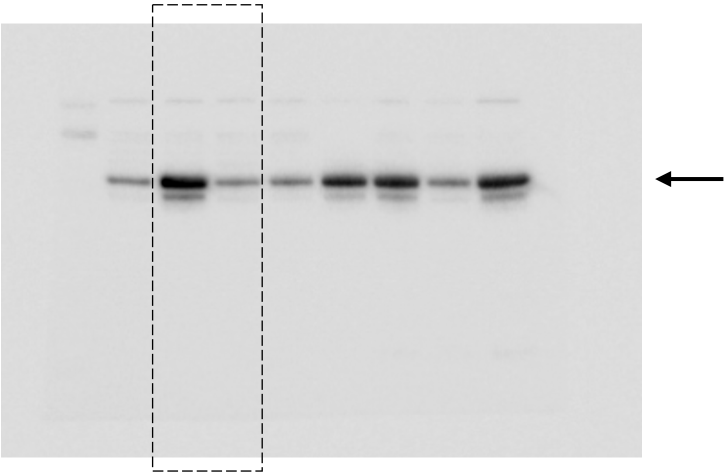

LN2E1 β-ACTIN:

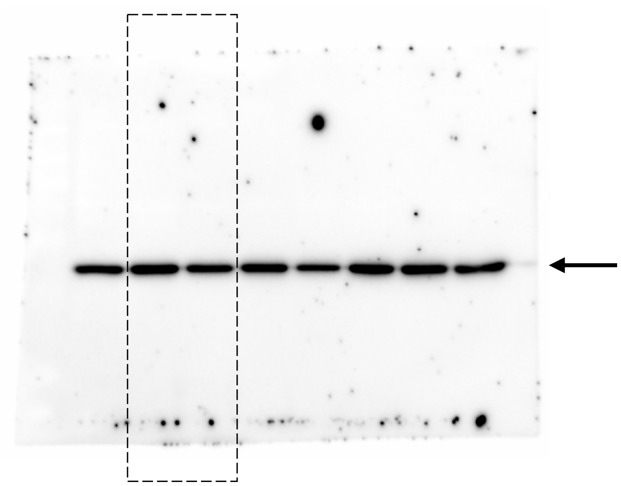

Full unedited blot for Supplementary Figure 6E:

LRP8:

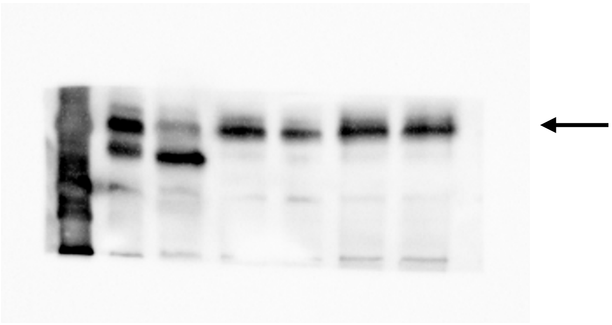

VINCULIN:

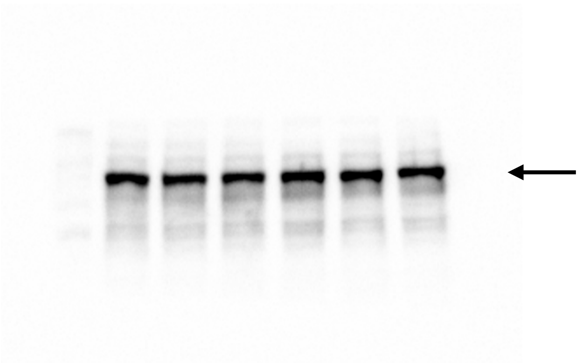

Full unedited blot for Supplementary Figure 7B:

β-ACTIN:

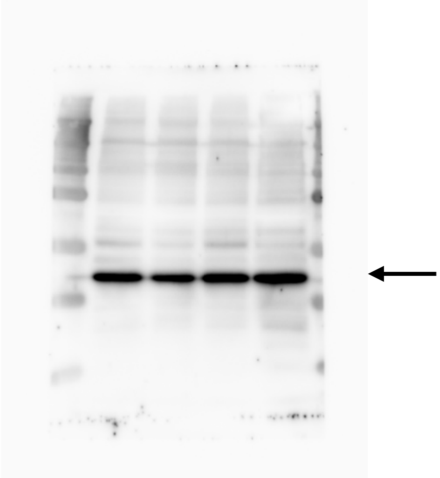

pSMAD2:

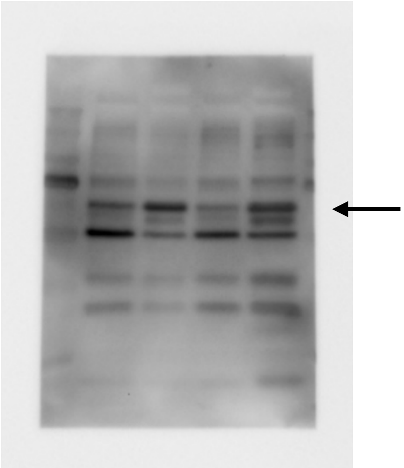

total SMAD2:

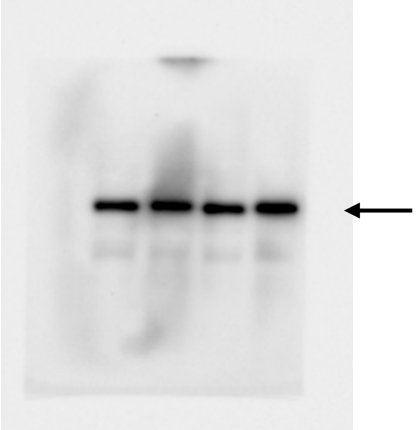

Full unedited blot for Supplementary Figure 7D:

VINCULIN:

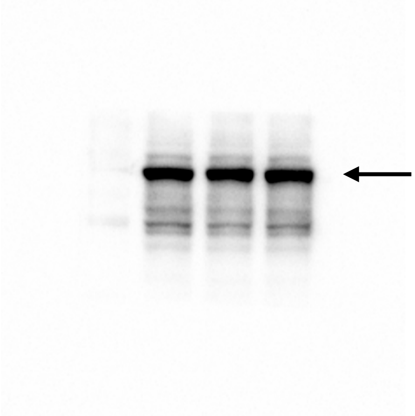

pSMAD2:

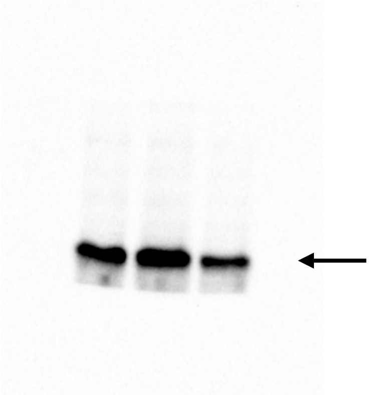

total SMAD2:

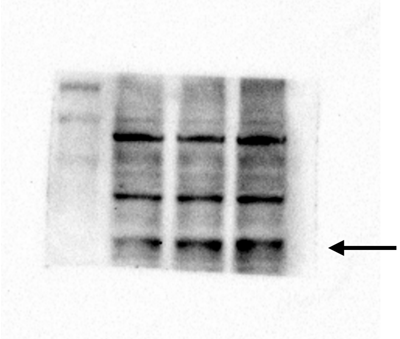

Supplement: Unedited blot and gel images [file jciinsight-9-168792-s160.pdf]
